# MLPA and DNA index improve the molecular diagnosis of childhood B-cell acute lymphoblastic leukemia

**DOI:** 10.1038/s41598-020-68311-9

**Published:** 2020-07-13

**Authors:** Chih-Hsiang Yu, Tze-Kang Lin, Shiann-Tarng Jou, Chien-Yu Lin, Kai-Hsin Lin, Meng-Yao Lu, Shu-Huey Chen, Chao-Neng Cheng, Kang-Hsi Wu, Shih-Chung Wang, Hsiu-Hao Chang, Meng-Ju Li, Yu-Ling Ni, Yi-Ning Su, Dong-Tsamn Lin, Hsuan-Yu Chen, Christine J. Harrison, Chia-Cheng Hung, Shu-Wha Lin, Yung-Li Yang

**Affiliations:** 1 Department of Clinical Laboratory Sciences and Medical Biotechnology, College of Medicine, National Taiwan University, Taipei, Taiwan; 2 Graduate Institute of Clinical Medicine, National Taiwan University, Taipei, Taiwan; 3 Sofiva Genomics Co., Ltd, Taipei, Taiwan; 4 Department of Pediatrics, National Taiwan University Hospital and National Taiwan University College of Medicine, Taipei, Taiwan; 5 Institute of Statistical Science Academia Sinica, Taipei, Taiwan; 6 Department of Pediatrics, Taipei Medical University-Shuang Ho Hospital, Taipei, Taiwan; 7 Department of Pediatrics, National Cheng Kung University Hospital, Tainan, Taiwan; 8 Division of Pediatric Hematology and Oncology, China Medical University Children’s Hospital, Taichung, Taiwan; 9 Department of Pediatrics, Changhua Christian Hospital, Changhua, Taiwan; 10 Department of Pediatrics, National Taiwan University Hospital Hsin-Chu Branch, Hsinchu, Taiwan; 11 Department of Laboratory Medicine, National Taiwan University Hospital, Taipei, Taiwan; 12 Leukaemia Research Cytogenetics Group, Northern Institute for Cancer Research, Newcastle University, Newcastle-upon-Tyne, UK; 13 Department of Laboratory Medicine, College of Medicine, National Taiwan University, Taipei, Taiwan

**Keywords:** Medical research, Molecular medicine

## Abstract

Aneuploidy occurs within a significant proportion of childhood B-cell acute lymphoblastic leukemia (B-ALL). Some copy number variations (CNV), associated with novel subtypes of childhood B-ALL, have prognostic significance. A total of 233 childhood B-ALL patients were enrolled into this study. Focal copy number alterations of *ERG*, *IKZF1*, *PAX5*, *ETV6*, *RB1*, *BTG1*, *EBF1*, *CDKN2A*/*2B*, and the Xp22.33/Yp11.31 region were assessed by Multiplex Ligation-dependent Probe Amplification (MLPA). The MLPA telomere kit was used to identify aneuploidy through detection of whole chromosome loss or gain. We carried out these procedures alongside measurement of DNA index in order to identify, aneuploidy status in our cohort. MLPA telomere data and DNA index correlated well with aneuploidy status at higher sensitivity than cytogenetic analysis. Three masked hypodiploid patients, undetected by cytogenetics, and their associated copy number neutral loss of heterozygosity (CN-LOH) were identified by STR and SNP arrays. Rearrangements of *TCF3*, located to 19p, were frequently associated with 19p deletions. Other genetic alterations including iAMP21, *IKZF1* deletions, *ERG* deletions, *PAX5*^AMP^, which have clinical significance or are associated with novel subtypes of ALL, were identified. In conclusion, appropriate application of MLPA aids the identifications of CNV and aneuploidy in childhood B-ALL.

## Introduction

As the most common pediatric cancer, acute lymphoblastic leukemia (ALL) accounts for approximately 25% of childhood malignancies. With improved risk-directed treatment and supportive care, the overall 5-year event-free survival rates for this disease now exceed 80% in developed countries^1–3^. The two major features of risk-directed therapy are based on the genetic alterations of the leukemic cells at diagnosis and the determination of initial treatment response (measured by minimal residual disease, MRD, after induction therapy). The interpretation of MRD levels depends upon the subtype of ALL^4,5^. Although karyotyping has been the most common approach for detection of numerical chromosomal changes, molecular methods may enhance their detection in childhood B-ALL. Multiplex Ligation-dependent Probe Amplification (MLPA) is a sensitive method based upon the multiplex polymerase chain reaction and capillary electrophoresis that detects multiple copies of around 50 different genomic DNA targets. It has the advantage of lower price and quicker turn-around time than DNA arrays for identification of the important genetic alterations and is now widely used for detection of the important copy number changes in ALL^6,7^.

Gain or loss of whole chromosomes (aneuploidy) and intrachromosomal amplification of chromosome 21 (iAMP21) accounts for almost 30% of childhood B-ALL identified by traditional methods. High hyperdiploidy with greater than 50 chromosomes comprises up to 30% of childhood B-ALL and most commonly involves gains of chromosomes X, 4, 10, 14, 17 and 21^8^. It is associated with a good outcome, even in patients with induction failure^9^. Hypodiploidy with less than 44 chromosomes is less common (found in approximately 3% of cases) and is associated with an inferior outcome. Hypodiploid B-ALL can be further divided into three subgroups according to chromosome number. The most common are near-haploidy with 24–31 chromosomes and low-hypodiploidy with 32–39 chromosomes. High-hypodiploidy with 40–43 chromosomes is rare. Low-hypodiploid ALL has a high incidence of *TP53* germline mutations^10^. DNA index (DI) is a well-established method for detection of high hyperdiploidy. The MLPA telomere kit identifies specific gain or loss of individual chromosomes and is suitable for screening for whole chromosome numerical changes^11,12^. Masked low hypodiploidy, manifesting as doubling of the low hypodiploid chromosome number, can be difficult to diagnose^13^. Here we show that MLPA and DI are useful in its detection, as confirmed by single-nucleotide polymorphism (SNP) arrays and short tandem repeats (STR). B-ALL patients with iAMP21-ALL were initially shown to have a high relapse risk on standard chemotherapy^14,15^. It was later demonstrated that treatment on intensive therapy regimens significantly reduced their risk of relapse^16–18^.

In childhood B-ALL, SNP arrays have successfully identified copy number abnormalities (CNA)^19^ involving several signaling pathways. For example, deletions of a number of genes within the B-cell differentiation pathway were identified, including *PAX5*, *EFB1* and *IKZF1*^19,20^. Clinically, *IKZF1* alterations have been associated with a poor outcome, particularly in association with Ph-positive (Philadelphia chromosome/*BCR-ABL1* positive), and Ph-like ALL (Philadelphia chromosome/*BCR-ABL1* negative but the expression profiles were similar to Ph-positive ALL)^20–27^. Ph-like and iAMP21-ALL have been proposed as novel subtypes of B-ALL in the recent WHO classification of hematologic malignancies, due to their poor prognostic associations^28^.

In this project, we have used MLPA and DI to study CNA in B-ALL. We show that these approaches are complementary to cytogenetics in improving detection of genetic alterations in childhood B-ALL.

## Materials and methods

### Patients and protocols

Diagnostic bone marrow (BM) or peripheral blood was obtained from 233 children with B-ALL from January 2002 to July 2018 at the National Taiwan University Hospital. A total of 108 patients were treated on the Taiwan Pediatric Oncology Group TPOG-ALL-2002 protocol, while 125 were treated on TPOG-ALL-2013. Diagnosis of B-ALL was based on BM morphology and the immunophenotype of leukemic cells was determined by flow cytometry. Conventional cytogenetic analysis was carried out as part of the routine work-up^29^.

The risk-directed TPOG protocols consist of multiple chemotherapeutic agents of different intensities. The treatment protocol was intensified if complete remission was not achieved after initial induction therapy. After 2013, MRD levels were added to risk assignment for therapy. Events were defined as any relapse, death, or secondary malignancy. The Institutional Review Board of National Taiwan University Hospital approved the study and all of the participants or their guardians provided written informed consent in accordance with the Declaration of Helsinki. Details of the protocols and risk group assignment have been published elsewhere^27,30,31^. We have summarized the risk classification of protocols in the Supplementary information.

### Genomic DNA extraction

Lymphoblasts were purified from bone marrow or peripheral blood specimens using the Ficoll-Paque centrifugation method, according to the manufacturer’s instructions (GE Healthcare, Piscataway, NJ, USA). Genomic DNA was extracted from leukemic cells using standard phenol/chloroform-based methods. Briefly, 1 million cells were lysed in 10 mM Tris–HCl, 10 mM NaCl, 10 mM EDTA, 20 μg proteinase K, and 0.5% SDS by incubating at 37 °C for 16 h. Total RNA was further removed by adding 500 μg PureLink RNase A (Invitrogen, USA) and incubating for 10 min at 37 °C. An equal volume of phenol–chloroform–isopropanol (25:24:1) was added to lysates and mixed by shaking vigorously, followed by centrifugation at 16,100 × *g* at 4 °C for 5 min. The upper aqueous phase was transferred to a fresh tube; genomic DNA was then precipitated by adding 2× volume − 80 °C 100% ethanol. The DNA pellet was washed with 75% ethanol and rehydrated with Tris–EDTA buffer. The concentration of DNA was determined using a NanoDrop 1,000 spectrophotometer (Thermo Fisher Scientific, Waltham, Massachusetts, USA)^32^.

### MLPA analysis

Genomic DNA was analyzed using the SALSA MLPA kit (MRC-Holland, Amsterdam, the Netherlands), according to manufacturer’s instructions. The PCR fragments were separated by capillary electrophoresis on a Life Technologies 3,500 Genetic Analyzer (Thermo Fisher Scientific, Waltham, MA, USA). MLPA data were analyzed using Coffalyser.Net v.140721.1958 (MRC-Holland, Amsterdam, The Netherlands). Probe ratio between 0.75 and 1.3 were considered to be within the normal range. Probe ratio below 0.75 or above 1.3 indicated deletion or gain, respectively. Probe ratio below 0.25 or above 1.8 indicated biallelic deletion or amplification. SALSA MLPA P335 ALL-IKZF1 probemix was used for detection of alterations of *EBF1*, *IKZF1*, *CDKN2A*, *CDKN2B*, *PAX5*, *ETV6*, *RB1* and *BTG1* genes. SALSA MLPA P327 iAMP21-ERG probemix was used for detecting alterations of *ERG* gene and iAMP21. SALSA MLPA P329 CRLF2-CSF2RA-IL3RA probemix was used for detecting *P2RY8-CRLF2* (PAR1 deletion).

### Analysis of ploidy status

Ploidy status was evaluated by SALSA MLPA P036 Subtelomeres Mix 1 probe mix. Whole chromosomal gain or loss was defined when two probes targeting p and q arms of the same chromosome were respectively gained or deleted simultaneously. Chromosome 19p deletions were defined when the probe targeted the p arm of chromosome 19 was deleted while q arm was normal.

### DNA index measured by flow cytometry

Freshly prepared or frozen leukemia samples were used for DNA index analysis. Peripheral blood derived from normal healthy individuals was used as controls for diploidy. Mononuclear cells were isolated by Ficoll-Paque (GE Healthcare, Chicago, IL, USA) according to the manufacturer’s instructions. Three cell suspensions were prepared: tube A was a mixture of leukemia cells and normal PBMCs in equal numbers; tubes B and C contained normal PBMCs or leukemia cells alone. Each cell suspension (2 million cells) was fixed with 70% ethanol overnight at – 20 °C. Fixed cells were washed with 1× PBS and then incubated with propidium iodide (50 μg) and RNase (10 μg) for 1 h on ice. Cells were filtered with 100 μm cell strainer and then analyzed by FACSCalibur (BD, Franklin Lakes, NJ, USA). DNA quantity of an individual cell population was determined and DNA index represents the ratio of leukemia sample/normal PBMCs fluorescence calculated from tube A. Tubes B and tube C were used as reference to distinguish the leukemia from PBMC peaks in tube A. Theoretical DNA index (tDI) was calculated using the formula: tDI = chromosome numbers × 0.0202 + 0.0675^33^.

### Statistical analysis

Pearson's correlations, the coefficient of determination and p-values were carried out between the results of DI and tDI from MLPA and cytogenetics. Fisher’s exact test was performed to evaluate the enrichment of 19p deletion in *TCF3* gene rearranged ALL. The log-rank test compared different survival curves between patients with different major genetic subtypes, patient with or without *IKZF1* deletion and patients with or without *IKZF1*^plus^. Overall survival (OS) was defined as diagnosis to death. Patients who did not suffer any adverse events within the follow-up period were censored. Event-free survival (EFS) of patients with no response to chemotherapy (refractory), death, and second relapse in induction was set to 0. Univariate and multivariate Cox regression were performed to evaluate hazard ratios (HR) and 95% confidence intervals (CI) of risk factors. All statistical analyses were performed using the Statistical Product and Services Solutions (SPSS) statistical package, v18.0 (IBM, Armonk, NY, USA).

## Results

### Frequency of copy number abnormalities in children with B-ALL

The demographic, clinical, and laboratory characteristics of 233 children with B-ALL are shown in Table 1. The median age of the cohort was 5.4 years (range < 0.1–17.9 years); 95.3% of the patients were over 1 year of age. The molecular tests performed were those standardized for B-ALL diagnosis including: *ETV6-RUNX1*, *TCF3-PBX1*, *BCR-ABL1*, *P2RY8-CRLF2* and *KMT2A-AFF1* for 220 samples. Detailed flow diagram of analysis used in this study is demonstrated in Supplementary Fig. S1.

**Table 1:** The characteristics of patients in this cohort.

	N	%
Sample size	233	100
**Gender**		
< 1 year	11	4.7
1–9 years	160	68.7
> 10 years	62	26.6
**Sex**		
Female	113	48.5
Male	120	51.5
**WBC (× 10**^**9**^**/l)**		
< 50	164	70.4
50–100	28	12.0
> 100	38	16.3
No data	3	1.3
**Protocol**		
TPOG 2002	108	46.4
TPOG 2013	125	53.6
**Early treatment response**^a^		
MRD positive	30	27.0
MRD negative	81	73.0
**Subtype**		
Hyperdiploidy	59	25.3
Hypodiploidy	7	3.0
*ETV6-RUNX1*	36	15.5
*TCF3-PBX1*	12	5.2
Ph+/Ph-like	16	6.9
*KMT2A-r*	14	6.0
*ZNF384/362-r*	9	3.9
*MEF2D-r*	3	1.3
*TCF3-HLF*	2	0.9
iAMP21	4	1.7
Other	71	30.5

^a^Only patients treated with TPOG-2013 were included (n = 111). Fourteen patients were excluded due to missing data; MRD positive: either MRD > 1% at day 15 or > 0.01% at day 35.

**Figure 1:**
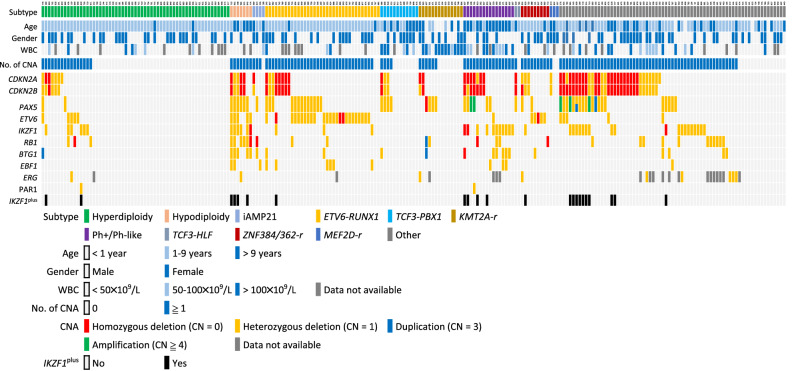
The heatmap of major subtype of childhood B-cell ALL and the association with CNA detected by MLPA. *Ph+* *BCR-ABL1* positive, *No.* number, *CN* copy number.

From MLPA testing, overall, 65.7% of the patients (153/233) harbored at least one abnormality (either deletion or amplification) involving the following genes—*IKZF1*, *CDKN2A*/*2B*, *PAX5*, *EBF1*, *ETV6*, *BTG1*, *RB1*, *ERG* or PAR1 region, whereas the remaining 34.3% (80/233) of patients had none of these abnormalities. Simultaneous aberrations in different genes were observed. A heatmap listing these CNA in the entire cohort are given in Fig. 1. Details of the CNA in each major cytogenetic subtype are shown in Supplementary Table S2.

### DNA index identifies ploidy status in ALL

In 112 samples DNA index analysis was performed; 41 cases showed aneuploidy, of which 35 were high hyperdiploid, 3 were hypodiploid and in 3 cases masked hypodiploidy was indicted, as described below. However, DI cannot identify individual chromosome gain or loss.

### MLPA compared to DI and cytogenetics

**Figure 2:**
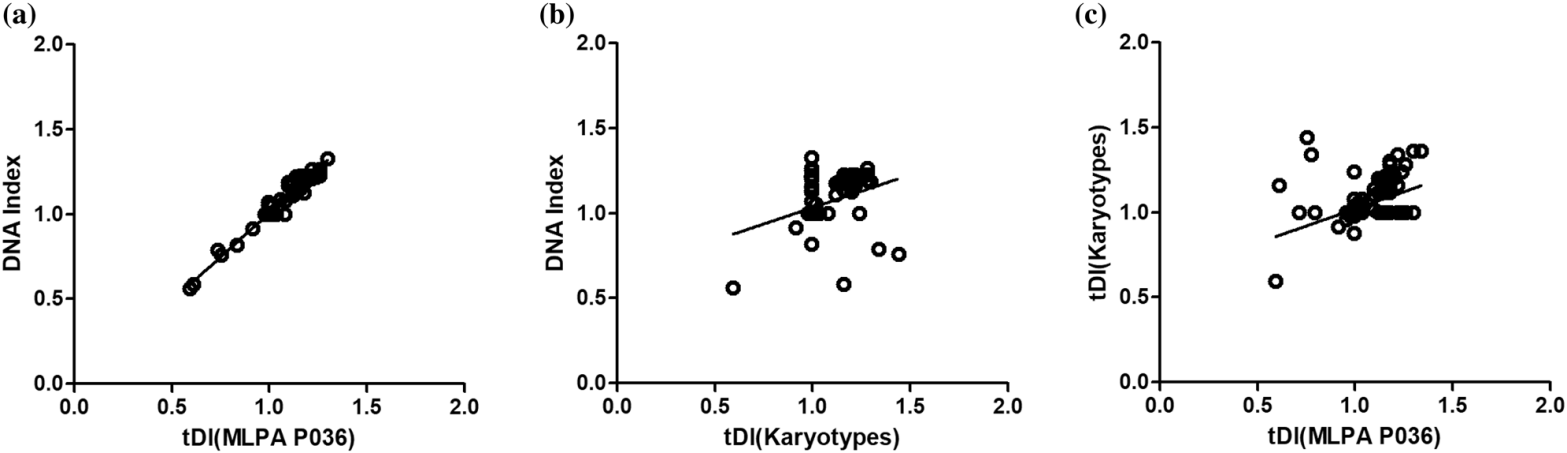
Correlation of the DI and tDI from MLPA P036 and karyotype. (**a**) DI vs. tDI(MLPA P036) (r = 0.9780, P < 0.0001). (**b**) DI vs. tDI (Karyotypes) (r = 0.3308, P = 0.0005). (**c**) tDI (Karyotypes) vs. tDI (MLPA P036) (r = 0.4428, P < 0.0001). *tDI* theoretical DNA index.

Good quality genomic DNA was available from 204 samples for MLPA analysis using the MLPA P036 kit which identified 57 patients with high hyperdiploidy, 7 with hypodiploidy and 140 with diploidy or near-diploidy. The numerical chromosomal alterations determined by this MLPA P036 kit were compared with the karyotype and DI results. These results showed concordance in number of chromosomes (r = 0.9780, P < 0.0001) for the 111 patients with both MLPA and DI data available (Fig. 2a). There was statistically significant positive correlation between karyotype and DI (r = 0.3308, P = 0.0005) (Fig. 2b), yet lower than MLPA against DI, among 188 patients with karyotype and MLPA data available. The statistically significant positive correlation was also seen between karyotype and MLPA (r = 0.4428, P < 0.0001) (Fig. 2c), but lower than MLPA against DI. We found that 45% (29/64) of patients with high hyperdiploidy or hypodiploidy identified either by DI or MLPA P036 were non-informative. Details of karyotype, DI and MLPA of the cohort are listed in Supplementary Table S3.

### High hyperdiploidy

Among 57 cases with high hyperdiploidy, the majority (94.5%) had gained between 5 and 13 chromosomes (modal chromosome number, MCN, 51–63, inclusive), and the most frequent MCN was 54 chromosomes (Supplementary Fig. S2a). Chromosome gains were non-random and 8 chromosomes accounted for 82% of all gains: 4 (72.7% of cases), 6 (80.7%), 10 (84.2%), 14 (93.0%), 17 (80.7%), 18 (86.0%), 21 (100%), and X (78.9%) (Supplementary Fig. S2b). Gains of chromosomes 5, 8, 9, 11, 12, and 22 represented 15% of the total and were present in between 11 and 35% of cases. Gains of chromosomes 1, 2, 3, 7, 13, 15, 16, 19, and 20 were rare, totaling 3% of chromosomes gained. These patterns of chromosomal gains in these high hyperdiploid cases were similar to previous reports.

### The MLPA pattern of iAMP21 and differentiation between iAMP21 and high hyperdiploidy

From their MLPA plots, we identified four patients with iAMP21, as shown in Supplementary Fig. S3. A characteristic chromosome 21 copy number profile has been previously described for cases of iAMP21-ALL from microarray studies and next generation sequencing. It is described as copy number changes from centromere to telomere along chromosome 21, with the highest level of amplification proximal to a telomeric deletion^34–36^. Tsuchiya et al. reported a case in which *RUNX1* was not located within the highest region of amplification of chromosome 21^37^. In this cohort, *RUNX1* was observed within the most highly amplified region of chromosome 21, with the exception of one case (Supplementary Fig. S3). In high hyperdiploid cases, the DI is usually greater than 1.16 and associated with frequent gains of chromosomes 4, 6, 10, 14, 18, 21 and X. We compared the pattern of chromosome 21 gain in high hyperdiploid and iAMP21-ALL in our cohort. SNP arrays analysis was carried out on two iAMP21-ALL samples diagnosed by MLPA (Supplementary Fig. S4). For cases with suspected iAMP21, in the absence of SNP arrays, DI and MLPA P036 and P327 kits can provide the definitive answer.

### Hypodiploid cases

**Figure 3:**
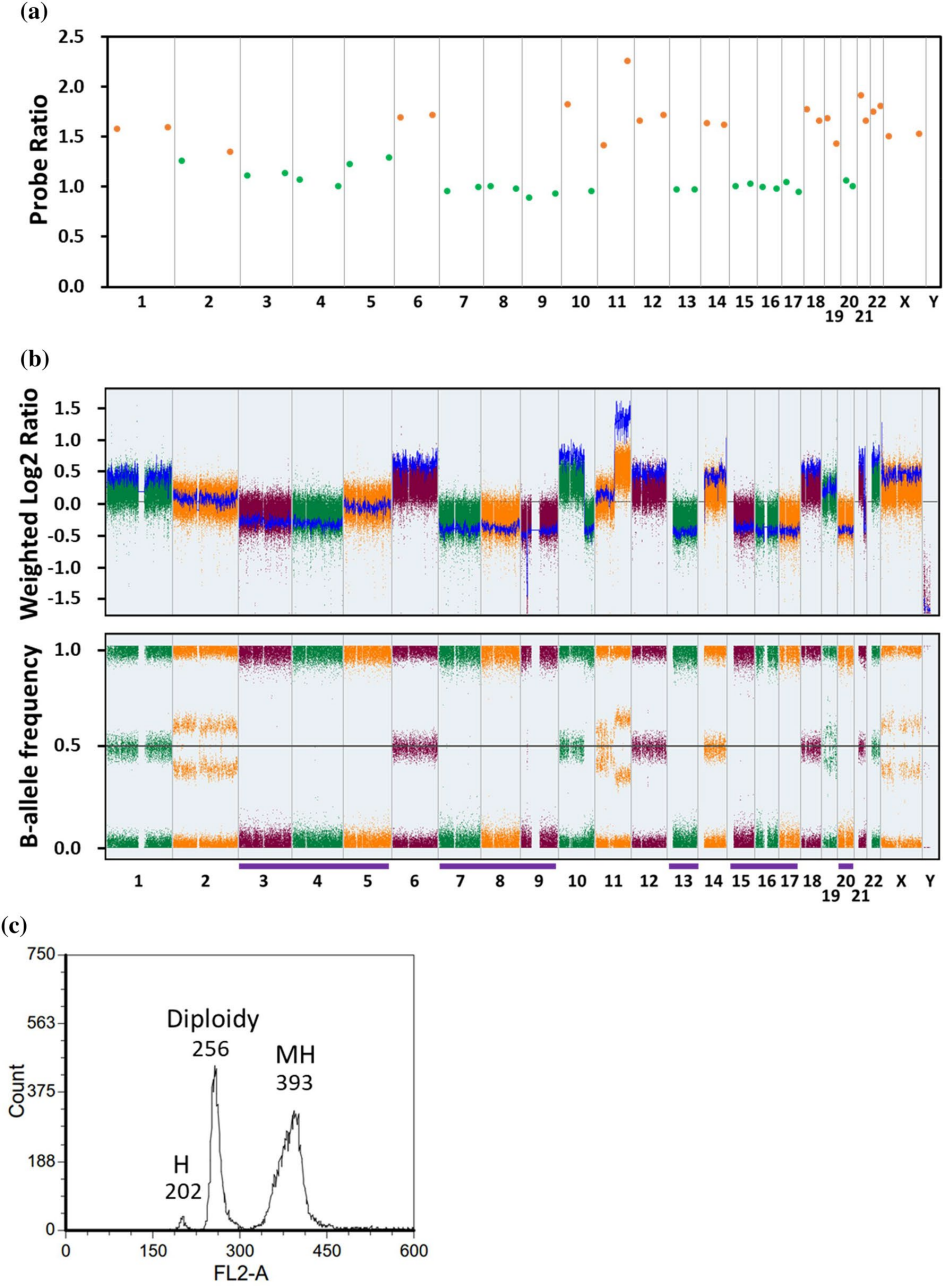
Analysis of a case with masked hypodiploidy. (**a**) The result of MLPA P036. The probe ratio values between 0.75 and 1.3 or greater than 1.3 were indicated with green or orange dots respectively. (**b**) The result of CytoScan array. Weight log2 ratio and B-allele frequency (BAF) plots are shown. The BAF plot show only two tracts indicate a region with LOH and the whole chromosome LOH is indicated by purple lines. (**c**) The result of DNA index analysis. *H* hypodiploidy, *MH* masked hypodiploidy.

Five patients with low DI were diagnosed with hypodiploidy. Three of them had two peaks in the DI, indicating the presence of hypodiploid clone undergo a doubling of the chromosomes during metaphase. This manifestation is known as masked hypodiploidy. As the diagnosis of masked hypodiploidy requires demonstration of loss of heterozygosity (LOH), these three samples were analyzed by SNP arrays and LOH was seen, as shown in case 984 (Fig. 3). DI showed two peaks: the smaller one (FL2-A value = 202) is the true hypodiploidy and the higher one (FL2-A value = 393) indicates the doubled hypodiploid population. These hypodiploid samples were also tested using MLPA P036 kit. By comparing MLPA with the value of DI, we were able to identify the specific losses and retention of each chromosome number. Thus, we were able to confirm that the masked hypodiploid population originated from doubling of the low hypodiploid one. In Fig. 3, the chromosome gains detected by MLPA P036 corresponded to the retained chromosomes. In contrast, the “normal” chromosomes, for example chromosomes 3, 4, 5, 7, 8, 9, 13, 15, 16, 17 and 20 were shown to be lost. The actual gain or loss of each chromosome cannot be inferred from the DNA index. Using the MLPA P036 kit, we identified another two cases of hypodiploidy (patients 508 and 753) in which LOH was confirmed by STR (see below). Details of these patients are listed in the Table 2.

**Table 2:** Detailed information of hypodiploidy cases.

ID	Subtype	Karyotype	MLPA P036	DNA index	*TP53* mutation
925	High-hypodiploidy	42,X,-4,-9,-13,t(14;17)(q32;p11.2),add(21)(p11.2)[16]/84,idem × 2[3]/46,XY[1]	42,Y,-4,-9,-13	0.91	R248Q (Unknown)
774	Low-hypodiploidy	46,XY[25]	38,XY,-2,-3,-12,-13,-14,-15,-16,-17	0.82	F341fs (Somatic)
508	Low-hypodiploidy	46,XY[20]	32,XY,-2,-3,-4,-6,-7,-9,-10,-12,-15,-16,-17,-18,-20,-22	NA	I195F (Germline)
984	Masked Low-hypodiploidy	63,XX,-X, + 1,-3,add(3)(q13),del(3)(q?21),-4,-5, + 6,-7,-9,-9,-10, + 12,-13, + 14,-15,-16,-17,-20, + add(21)(q22) × 2, + 22, + 2mar,inc[cp14]/46,XX[6]	33,XX,-3,-4,-5,-7,-8,-9,-10,-11,-13,-15,-16,-17,-20	0.79/1.54	R273H (Germline)
845	Masked Low-hypodiploidy	68,XX,-Y, + 1,-2,-3,-4,del(4)(q21q31),del(5)(q13q33), + 6,-7, + 8, + 9,-10, + 11,-12,-13, + 14,-15,-16,-17,-18, + 19, + 20, + 21, + 22,inc[cp5]/46,XY[20]	34,Y,-2,-3,-4,-7,-10,-12,-13,-15,-16,-17,-18	0.76/1.43	W53* (Somatic)
952	Near-haploidy	26,XY,-1,-2,-3,-4,-5,-6,-7,-8,-9,-11,-12,-13,-14,-15,-16,-17,-18,-19,-20,-22[13]	26,XY,-1,-2,-3,-4,-5,-6,-7,-8,-9,-11,-12,-13,-14,-15,-16,-17,-18,-19,-20,-22	0.56	Wild-type
753	Masked Near-haploidy	52–54,XX, + mar1 ~ mar8[cp4]/46,XX[21]	27,X,-1,-2,-3,-5,-6,-7,-9,-10,-11,-12,-13,-15,-16,-17,-18,-19,-20,-22	1.17	Wild-type

A Short Tandem Repeat (STR) is a microsatellite, consisting of a unit of two to thirteen nucleotides repeated hundreds of times on a DNA strand. STR analysis measures the precise number of repeating units. STR is used for confirmation of donor engraftment following stem cell transplantations and this test is available in all medical centers^38^. Samples of germline (if available) and tumor were sent for STR analysis in order to confirm LOH identified on SNP arrays. We show the interpretation of STR for patient 984 in Supplementary Fig. S5 and all three cases of masked hypodiploidy by STR are shown in Supplementary Table S4. STR provides a simple method to confirm the presence of LOH. Based upon these observations, we have proposed a flowchart for diagnosis of masked hypodiploidy (Supplementary Fig. S6).

### 19p deletion by MLPA is an indicator of *TCF3* translocations in childhood ALL

We identified 7 of 12 cases of *TCF3-PBX1* and two cases of *TCF3-HLF* with 19p loss. This enrichment differs from other subtypes of B-cell ALL (P < 0.0001) (Table 3). *TCF3* is an important transcriptional factor with multiple fusion partners in ALL. Samples with 19p deletions without evidence of *TCF3-PBX1* or *TCF3-HLF* fusions may carry *TCF3-ZNF384* fusions. *TCF3-ZNF384* fusions represent another important subtype of B-cell ALL with a specific immunophenotype showing frequent CD10 loss and CD13 and CD33 expression. From these observations, we observed one sample with 19p loss, loss of CD10, CD13 and CD33 expression in which the *TCF3-ZNF384* fusions was identified by RT-PCR (Supplementary Fig. S7). In this series, among a total of 15 samples with 19p loss, 10 of them had *TCF3* fusions.

**Table 3:** The 19p deletion in *TCF3* translocation and non-*TCF3* translocation subtypes.

	19p Normal	19p Deletion
***TCF3 translocation***	7	10
*TCF3-PBX1*	5	7
*TCF3-HLF*	0	2
*TCF3-ZNF384*	2	1
**Non-TCF3 translocation**	162	5
Hyperdiploidy	55	3
*ETV6-RUNX1*	22	1
*KMT2A* fusions	11	1
Other	74	0

Fisher’s exact test of comparing *TCF3* translocation with non-*TCF3* translocation subtypes, P < 0.0001.

### Novel subtypes of ALL, intragenic amplifications of *PAX5* (*PAX5*^AMP^), *IKZF1-plus* and *ERG* deletions

Recently two papers have reported two novel high-risk subtypes of childhood ALL, *PAX5*^AMP^ and *IKZF1*^plus^^7,39^. There were 23 *IKZF1*^plus^ patients and 5 patients with *PAX5*^AMP^ in this cohort. Nine patients (9/233 = 3.9%) were identified with *ERG* deletions. These *ERG* deletions were associated with different subtypes of ALL (Fig. 1).

### Survival analysis

**Figure 4:**
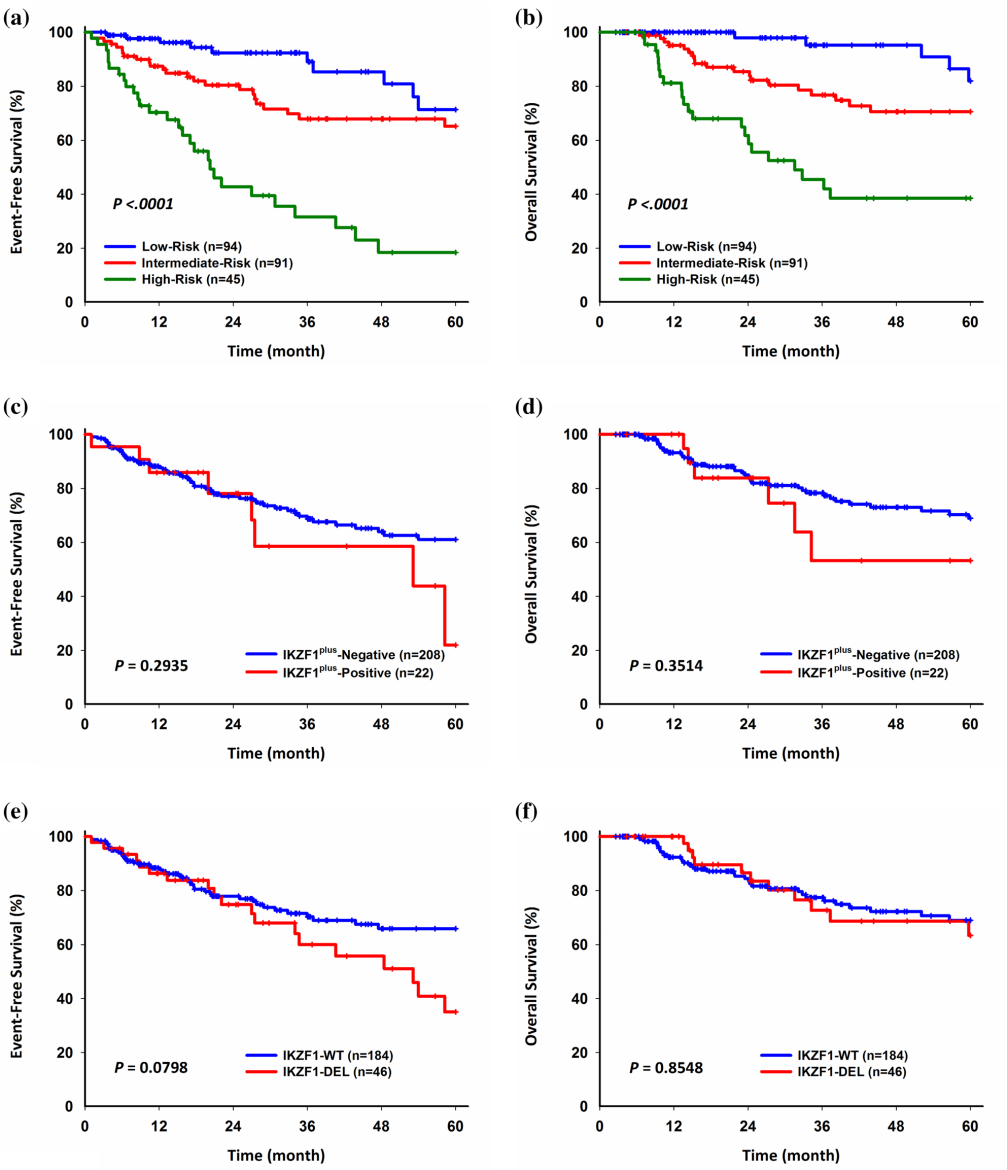
Five-year EFS and OS analysis. (**a**, **b**) Major genetic subtypes. (**c**, **d**) *IKZF1*^plus^-Positive vs. *IKZF1*^plus^-Negative. (**e**, **f**) *IKZF1*-DEL vs. *IKZF1*-WT. *EFS* event-free survival, *OS* overall survival, *DEL* deletion, *WT* wild type.

Among patients with the major cytogenetic alterations, two with *TCF3-HLF* relapsed and died within 5 years from diagnosis. Patients with high-risk subtypes (Ph-positive/-like, hypodiploidy, *MEF2D*-r, *KMT2A*-r, *TCF3-HLF*, iAMP21) had inferior 5-year EFS (P < 0.0001) and OS (P < 0.0001) (Fig. 4a, b). The overall outcome was slightly inferior compared to previous TPOG reports, likely due to many of them being referred from other hospitals after relapse^30^. All patients with iAMP21 were not detected at diagnosis. There is a trend that patients with *IKZF1*^plus^ had inferior 5 year-EFS and OS than patients without *IKZF1*^plus^, but it did not reach statistical significance (Fig. 4c, d). Patients with *IKZF1* deletions had inferior 5-year EFS and 5-year OS, but it also did not reach statistical significance (Fig. 4e, f). In the Cox multivariate regression model, *IKZF1* deletions were not a strong predictor of poor outcome (Supplementary Table S5).

## Discussion

In this retrospective study, the MLPA P036 subtelomeres probemix kit provided accurate detection of aneuploidy in childhood B-cell ALL and good correlation with the results from DI. MLPA and DI are superior to traditional cytogenetics, due to the shorter turn-around time, irrespective of mitotic index and improved sensitivity. Detections of specific gains or losses of each chromosome assist the differential diagnosis of hyperdiploidy from iAMP21. In addition, DI is helpful for diagnosis of masked hypodiploidy and LOH should be confirmed by SNP arrays. STR provides a simple method, available in most medical centers in Taiwan, to document LOH in these masked hypodiploid cases. Around 1.7% (4/233) of B-ALL patients had iAMP21. We also identified some of the novel ALL subtypes, including *PAX5*^AMP^, and *IKZF1*^plus^^7,39^. *TCF3* rearrangements were frequently associated with 19p deletions.

High hyperdiploidy accounts for around 20 ~ 25 percentage of childhood B-cell ALL^40^. In this cohort, the most frequent modal chromosome number was 54 followed by 55. The most frequent gains included chromosomes 4, 6, 10, 18, 16, 17, 18, 21 and X, in agreement with previous reports^8,40,41^. This incidence of high hyperdiploidy was lower in Taiwan than Caucasian populations^30,42,43^. Using DI and the MLPA P036 kit, the incidence was around 27% in this cohort. In this study, 45% of high hyperdiploid patients were not detected by cytogenetics, manifesting as normal karyotype. In previous TPOG ALL 2002 report, hyperdiploidy accounted for 13.6% in B-ALL (n = 1,209). The incidence was much lower than that of this report. The reason for this discrepancy might be the relative smaller case numbers in this study. For cases without metaphases or normal karyotype, DI and MLPA can be successfully used for diagnosis of high hyperdiploidy^11^.

iAMP21-ALL is a novel subtype of B-ALL proposed by WHO^14,15,28,33^. The initial gold standard for diagnosis was FISH using probes directed to the *RUNX1* gene, but array-CGH or SNP arrays are now the main method for diagnosis^33^. One MLPA kit can successfully identify iAMP21 due to the density of probes along the long arm of chromosome 21. We identified 4 cases with iAMP21 by MLPA. In these cases, the level of gain was variable along the length of chromosome 21 with the ratio being more than 3.0, higher than in cases where chromosome 21 is gained as part of a high hyperdiploidy karyotype in which the probe ratio for every probe in the kit being ~ 1.5–2.0. These data correlated with other gains, especially of chromosomes 4, 6, 10, 18, 16, 17, 18 and X. If gains of chromosomes X, 4, 6, 10, 14, 17 and 18 are detected at the same time as gains of 21, it is most likely that the patient has high hyperdiploidy rather than iAMP21-ALL.

Masked hypodiploidy can be difficult to diagnose. Another study used a similar MLPA approach to identify the aneuploidy status of relapsed B-cell ALL^12^. Three patients with high hyperdiploidy had the highest number of chromosomal gains (median 11). Gains of the classical high hyperdiploidy pattern were less frequent, but gains of non-classical chromosomes, especially 1, 5, 11, 19 and 22, accounted for 49% of all gains in these patients. All three patient relapse samples carried *TP53* mutations, two of which were present in the germline. In all three cases, no underlying hypodiploid clone was detected by DI or cytogenetic analyses, making diagnosis difficult. A recent report by Carroll et al. demonstrated that a considerable proportion (25% or higher) of hypodiploidy in children with B-ALL may have been overlooked in previous studies due to the presence of only a doubled hypodiploid population, mistakenly interpreted as typical high hyperdiploidy associated with a favorable risk^44^. In this cohort, the chromosome number in high hyperdiploidy was mostly in the range of 52 ~ 59, which could overlap with masked hypodiploidy. For masked hypodiploid cases, the MLPA P036 kit results, alongside DNA index, can detect the specific gain or loss of each chromosome. LOH can also be confirmed by STR.

*TCF3,* located to 19p, is rearranged with several genes in childhood ALL. The most frequent is *TCF3-PBX1* and rarely the poor risk *TCF3-HLF*^2^. We observed 19p loss in all *TCF3-PBX1* and *TCF3-HLF* cases. *TCF3* has also been identified to be rearranged with *ZNF384,* a novel fusion recently identified^45–48^. In cases with 19p deletions without *TCF3-PBX1* or *TCF3-HLF* detected by RT-PCR or cytogenetics, 19p deletions may point to other *TCF3* fusions. *TCF3-ZNF384* fusions are also frequently associated with CD10 loss, with the presence of CD13 and CD33^45,48,49^. These two characteristics are useful for its identification by RT-PCR.

In our cohort, patients with iAMP21 and *KMT2A* fusions had an inferior 5-year EFS and OS in comparison to patients with *ETV6-RUNX1* or high hyperdiploidy. Patients with hypodiploidy also had an inferior 5-year EFS and OS, although most of them were not identified at the time of diagnosis. The outcome for patients with iAMP21-ALL may be improved if detected at diagnosis, so that they may be treated with more intensive chemotherapy. No events were seen in patients with *PAX5*^AMP^, while patents with *IKZF1*^plus^ showed a trend towards inferior EFS and OS, although the *P*-value was not significant. *IKZF1* deletions showed a trend towards poorer clinical outcomes, as observed in a number of other studies^22,27,50^. Due to the relative small case numbers in this study, larger studies are indicated in Taiwan in order to evaluate the clinical impact of these genetic alterations in Taiwan.

In conclusion, MLPA and DNA index together can rapidly provide reliable information for identification of aneuploidy of childhood B-ALL. Using these methods, diagnosis of aneuploidy in Taiwan might be improved particularly among those cases currently classified within unknown subtype of B-cell ALL, and especially those without metaphases or normal karyotype. STR provides a simple method to demonstrate LOH if masked hypodiploidy is suspected. Other important abnormalities such as *IKZF1* deletions, *IKZF1*^plus^ and *ERG* deletions can also be identified by MLPA. These tools are helpful for the diagnosis of some important subtype of ALL.

## Supplementary information

Supplementary Information.
