# Supplementary material for: MLPA and DNA index improve the molecular diagnosis of childhood B-cell acute lymphoblastic leukemia
Source: Sci Rep. 2020 Jul 13;10:11501. doi: 10.1038/s41598-020-68311-9 (PMC7359332; doi:10.1038/s41598-020-68311-9)
Supplement: Supplementary file 1 — Supplementary Information. [file 41598_2020_68311_MOESM1_ESM.pdf]

Supplementary Information

MLPA and DNA index improve the molecular diagnosis of childhood B-cell acute lymphoblastic leukemia

Chih-Hsiang Yu[1], Tze-Kang Lin[2,3], Shiann-Tarng Jou[4], Chien-Yu Lin[5], Kai-Hsin Lin[4], Meng-Yao Lu[4], Shu-Huey Chen[6], Chao-Neng Cheng[7], Kang-Hsi Wu[8], Shih-Chung Wang[9], Hsiu-Hao Chang[4], Meng-Ju Li[4,10], Yu-Ling Ni[11], Yi-Ning Su[3], Dong-Tsamn Lin[4,11], Hsuan-Yu Chen[5], Christine J. Harrison[12], Chia-Cheng Hung[3*], Shu-Wha Lin[1*], Yung-Li Yang[4,11,13*]

[1]Departments of Clinical Laboratory Sciences and Medical Biotechnology, College of Medicine, National Taiwan University

[2]Graduate Institute of Clinical Medicine National Taiwan University, Taipei, Taiwan

[3]Sofiva Genomics Co., Ltd., Taipei, Taiwan

[4]Department of Pediatrics, National Taiwan University Hospital and National Taiwan University College of Medicine, Taipei, Taiwan

[5]Institute of Statistical Science Academia Sinica, Taipei, Taiwan

[6]Department of Pediatrics, Taipei Medical University–Shuang Ho Hospital, Taipei, Taiwan;

[7]Department of Pediatrics, National Cheng Kung University Hospital, Tainan, Taiwan

[8]Division of Pediatric Hematology &Oncology, China Medical University Children's Hospital, Taichung, Taiwan

[9]Department of Pediatrics, Changhua Christian Hospital, Changhua, Taiwan

[10]Department of Pediatrics, National Taiwan University Hospital Hsin-Chu Branch, Hsinchu, Taiwan

[11]Department of Laboratory Medicine, National Taiwan University Hospital, Taipei, Taiwan

[12]Leukaemia Research Cytogenetics Group, Northern Institute for Cancer Research, Newcastle University, Newcastle-upon-Tyne, United Kingdom

[13] Department of Laboratory Medicine, College of Medicine, National Taiwan University, Taipei, Taiwan

***Corresponding Authors**

Yung-Li Yang, Department of Laboratory Medicine, National Taiwan University Hospital and National Taiwan University College of Medicine, Taipei, Taiwan, 100, No 7. Chung-Shan South Road, (yangyl92@ntu.edu.tw), tel: +886-2-23123456-71712, fax: +886-2-23224263

Shu-Wha Lin, Departments of Clinical Laboratory Sciences and Medical Biotechnology, College of Medicine, National Taiwan University, Taipei, Taiwan, 100, No 7. Chung-Shan South Road, (mtshuwha@ntu.edu.tw), tel: +886-2-23123456-71712, fax: +886-2-23224263

Chia-Cheng Hung, Sofiva Genomics Co., Ltd., Taipei, Taiwan, No27, Baoqing Rd, Taipei, (double@sofivagenomics.com.tw), Taiwan, 100, tel: +886-2-23826615, fax: +886-2-23826617

Supplementary materials and methods
Supplemental references

Supplementary Table S1. Primer sequences
Supplementary Table S2. Distribution of CNA in each major B-ALL subtypes.
Supplementary Table S3. Detailed subtype, karyotype, MLPA P036 and DNA index results of B-ALL in this study.
Supplementary Table S4. STR profiling of masked hypodiploidy and high hyperdiploidy B-ALL cases.
Supplementary Table S5. 5-year EFS and OS using univariate and multivariate survival analysis.

Supplementary Figure S1. Flow diagram of analysis through this study.
Supplementary Figure S2. Analysis of high hyperdiploidy cases.
Supplementary Figure S3. The MLPA P327 of iAMP21-ALL.
Supplementary Figure S4. The results of CytoScan HD (Pt689) or 750K (Pt813) array.
Supplementary Figure S5. STR profiling for masked hypodiploidy.
Supplementary Figure S6. Flowchart for distinguishing masked hypodiploidy from high hyperdiploidy.
Supplementary Figure S7. Analysis of a case with *TCF3-ZNF384* case.

Supplementary materials and methods

Protocols

Patients were prospectively assigned to one of three risk groups (standard, high, and very high) based on their presenting clinical features and biology of the leukemic cells on the TPOG-ALL-2002 protocol. Patients were considered to have standard-risk (SR) ALL if they were between 1 and 9 years of age presenting with a leukocyte count of less than 10×10^9 cells/L, or were between 2 and 7 years of age presenting with a leukocyte count between 10×10^9 and 50×10^9 cells/L. Patients were considered to have high-risk ALL if they were between 1 and 9 years of age presenting with a leukocyte count between 50×10^9 and 100×10^9 cells/L, or between 1 and 2 or 7 and 10 years of age presenting with a leukocyte count between 10×10^9 and 50×10^9 cells/L. In addition, those with CNS leukemia or cranial nerve palsy at diagnosis, and those with central nervous system leukemia (cerebrospinal fluid white blood cells with blasts), cranial nerve palsy, testicular leukemia, or B-ALL with *TCF3-PBX1* fusion were also considered to be at high risk. Patients with at least one of the following were assigned to the very high-risk (VHR) group: age below 1 year, initial leukocyte count greater than 100×10^9 cells/L, lymphoblastic lymphoma with more than 25% lymphoblasts in the bone marrow, hypodiploidy, HR patients with poor treatment response, and the presence of *BCR-ABL1*, *KMT2A-AF4,* or other *KMT2A* rearrangements in B-ALL. The induction chemotherapy consisted of vincristine, epirubicin, prednisolone, L-asparaginase, cytarabine, cyclophosphamide, 6-mercaptopurine based upon the risk groups. Consolidation were used high dose methotrexate and 6-mercaptopurine. The drugs used in continuation phase including vincristine and dexamethasone, methotrexate, 6-mercaptopurine,

cytarabine, cyclophosphamide and etoposide depended upon the risk group. The details of drug administration were published elsewhere[1,2].

The risk classification system of TPOG-ALL-2013 is slightly different from that of 2002. B-lymphoblastic ALL with DNA index ≥ 1.16 [or hyperdiploidy (51-68)], *ETV6-RUNX1* fusion, or age 1 to 9.9 years and presenting WBC < 50,000/mm^3 and not have very high risk genetic alterations, poor early response. Patients with standard-risk included *ETV6-RUNX1* and hyperdiploidy. Criteria of very high-risk patients included *BCR-ABL1*, infant ALL, induction failure or ≥1% leukemic lymphoblasts in the bone marrow on remission date (with the exception of hyperdiploid (51-68) and *ETV6-RUNX1* cases who should have positive MRD after consolidation therapy), ≥0.1% leukemic lymphoblasts in the bone marrow in week 7 of continuation treatment. Other patients were classified as high-risk. The induction chemotherapy was similar to that of 2002, but adjusted by Day 15 MRD. High dose methotrexate and 6-mercaptopurine were used in the consolidation phase. The drugs used in continuation phase including vincristine and dexamethasone, methotrexate, 6-mercaptopurine, cytarabine, cyclophosphamide depended upon the risk group. Etoposide was no longer used in 2013 protocol.

Routine RT-PCR for common fusion gene

RNA obtained from BM or PB samples was isolated using standard methods.[3] Complement DNA (cDNA) was synthesized using Maxima First Strand cDNA Synthesis Kit (Thermo Fisher Scientific, Waltham, MA, USA). 1 μg total RNA was used for cDNA synthesis according to the manufacturer's

instructions. The prepared reaction mix was incubated for 10 minutes at 25℃, followed by 30 minutes at 60℃, then the reaction was terminated by heating at 85℃ for 5 minutes. For common fusion genes (*ETV6-RUNX1*, *KMT2A-AFF1*, *BCR-ABL1*, *TCF3-PBX1* and *P2RY8-CRLF2*) detection, MyTaq HS Mix (Bioline, London, United Kingdom) was used. Primers for RT-PCR used in this study are listed in Table S1. Thermocycling for PCR was performed: 98℃ for 30 seconds, then 38 cycles of 98℃ for 30 seconds, 60℃ for 30 seconds and 72℃ for 30 seconds, followed by final extension at 72℃ for 5 minutes.

Short tandem repeat (STR) profiling

Amelogenin and autosomal STR profiles were analyzed using VeriFiler Direct/Identifiler Direct Paternity Testing Bundle (Thermo Fisher Scientific, Waltham, MA, USA) according to the manufacturer protocol. Fragment analysis was carried on ABI 3730XL DNA Analyzer and automated profiling was performed using GeneMapper software 5 (Applied Biosystems, Waltham, MA, USA).

SNP arrays

The tumor DNA was used to detect chromosomal alterations by SNP array. Microarrays were performed using the CytoScan HD or 750K array kit (Affymetrix, Inc, Santa Clara, CA), according to the manufacturers' protocols and data were analyzed using Chromosome Analysis Suite (Affymetrix, Inc, Santa Clara, CA)

Transcriptome sequencing and bioinformatic analysis

After 2017, some newly or relapsed samples were submitted for RNA-seq analysis. RNA-seq was performed using TruSeq library preparation and HiSeq 2000 sequencer (Illumina, San Diego, CA, USA). All sequence reads were paired-end, and were performed by using total RNA-seq (100-base pair (bp) reads). All software was run using default parameters on a high-performance computing environment, and fastq files were mapped to the GRCh37 human genome reference by STAR v2.5.3a[4]. Gene annotation downloaded from the Ensembl website (http://www.ensembl.org/) was used for STAR mapping and the following read-count evaluation. FusionCatcher[5] were used to detect fusions, and all the reported rearrangements were manually reviewed and candidate fusion genes were validated by RT-PCR.

Supplemental references

1. Li M-J, Liu H-C, Yen H-J, et al: Treatment for childhood acute lymphoblastic leukemia in Taiwan: Taiwan Pediatric Oncology Group ALL-2002 study emphasizing optimal reinduction therapy and central nervous system preventive therapy without cranial radiation. Pediatric Blood & Cancer. **64**, 234-241 (2017).

2. Liang DC, Yang CP, Lin DT, et al: Long-term results of Taiwan Pediatric Oncology Group studies 1997 and 2002 for childhood acute lymphoblastic leukemia. Leukemia. **24**, 397-405 (2010).

3. Yang YL, Lin SR, Chen JS, et al: Multiplex reverse transcription-polymerase chain reaction as diagnostic molecular screening of 4 common fusion chimeric genes in Taiwanese children with acute lymphoblastic leukemia. J Pediatr Hematol Oncol. **32**, e323-30 (2010).

4. Dobin A, Davis CA, Schlesinger F, et al: STAR: ultrafast universal RNA-seq aligner. Bioinformatics. **29**, 15-21 (2012).

5. Nicorici D, Şatalan M, Edgren H, et al: FusionCatcher – a tool for finding somatic fusion genes in paired-end RNA-sequencing data. bioRxiv. 011650 (2014).

Supplementary Table S1. Primer sequences

Fusion gene	PCR primer (5’ to 3’)	
ETV6-RUNX1	Forward:	CGTGGATTTCAAACAGTCCA
	Reverse:	CATTGCCAGCCATCACAGTGAC
KMT2A-AFF1	Forward:	AAAGCAGCCTCCACCACC
	Reverse:	GGTTACAGAACTGACATGCTG
BCR-ABL1	Forward:	CGGTTGTCGTGTCCGAGG
	Reverse:	AGATACTCAGCGGCATTG
TCF3- PBX1	Forward:	CAGCCTCATGCACAACCAC
	Reverse:	TAACTCCTCTTTGGCTTCCTC
P2RY8-CRLF2	Forward:	GCGGCCGCCTTTGCAAGGTTGC
	Reverse:	GTGTCCATCACAACGCCACGTAGGA

Supplementary Table S2. Distribution of CNA in each major B-ALL subtypes.

	Total		Hyperdiploidy		Hypodiploidy		iAMP21		*ETV6-RUNX1*		*TCF3-PBX1*		*KMT2A* -r		Ph+/Ph-like		*TCF3-HLF*		*ZNF384/362* -r		*MEF2D* -r		Other	
	N	%	N	%	N	%	N	%	N	%	N	%	N	%	N	%	N	%	N	%	N	%	N	%
**Cases**	**233**	**100**	**59**	**100**	**7**	**100**	**4**	**100**	**36**	**100**	**12**	**100**	**14**	**100**	**16**	**100**	**2**	**100**	**9**	**100**	**3**	**100**	**71**	**100**
≥ 1 alterations	153	65.7	16	27.1	7	100	3	75.0	34	94.4	4	33.3	6	42.9	16	100	1	50.0	9	100	1	33.3	56	78.9
Without alterations	80	34.3	43	72.9	0	0	1	25.0	2	5.56	8	66.7	8	57.1	0	0	1	50.0	0	0	2	66.7	15	21.1
CDKN2A	**70**	**30.5**	**7**	**11.9**	**5**	**71.4**	**1**	**25.0**	**8**	**22.2**	**3**	**25.0**	**2**	**14.3**	**7**	**43.8**	**1**	**50.0**	**3**	**33.3**	**1**	**33.3**	**32**	**45.1**
Heterozygous deletion	28	12.4	5	8.5	2	28.6	0	0.0	2	5.6	2	16.7	0	0.0	1	6.3	0	0.0	3	33.3	1	33.3	12	16.9
Homozygous deletion	42	18.0	2	3.4	3	42.9	1	25.0	6	16.7	1	8.3	2	14.3	6	37.5	1	50.0	0	0.0	0	0.0	20	28.2
CDKN2B	**68**	**29.6**	**6**	**10.2**	**5**	**71.4**	**1**	**25.0**	**8**	**22.2**	**3**	**25.0**	**2**	**14.3**	**7**	**43.8**	**1**	**50.0**	**3**	**33.3**	**1**	**33.3**	**31**	**43.7**
Heterozygous deletion	27	12.0	5	8.5	2	28.6	1	25.0	2	5.6	2	16.7	1	7.1	1	6.3	0	0.0	2	22.2	1	33.3	10	14.1
Homozygous deletion	41	17.6	1	1.7	3	42.9	0	0.0	6	16.7	1	8.3	1	7.1	6	37.5	1	50.0	1	11.1	0	0.0	21	29.6
PAX5	**59**	**25.8**	**2**	**3.4**	**6**	**85.7**	**1**	**25.0**	**13**	**36.1**	**4**	**33.3**	**4**	**28.6**	**6**	**37.5**	**1**	**50.0**	**2**	**22.2**	**0**	**0.0**	**20**	**28.2**
Heterozygous deletion	51	22.3	2	3.4	6	85.7	1	25.0	13	36.1	4	33.3	3	21.4	4	25.0	1	50.0	2	22.2	0	0.0	15	21.1
Homozygous deletion	1	0.4	0	0.0	0	0.0	0	0.0	0	0.0	0	0.0	1	7.1	0	0.0	0	0.0	0	0.0	0	0.0	0	0.0
Exon 1-6 amplification and Heterozygous deletion	1	0.4	0	0.0	0	0.0	0	0.0	0	0.0	0	0.0	0	0.0	0	0.0	0	0.0	0	0.0	0	0.0	1	1.4
Exon 2-5 amplification	5	2.1	0	0.0	0	0.0	0	0.0	0	0.0	0	0.0	0	0.0	2	12.5	0	0.0	0	0.0	0	0.0	3	4.2
Exon 8-10 duplication	1	0.4	0	0.0	0	0.0	0	0.0	0	0.0	0	0.0	0	0.0	0	0.0	0	0.0	0	0.0	0	0.0	1	1.4
ETV6	**46**	**20.2**	**5**	**8.5**	**5**	**71.4**	**0**	**0.0**	**25**	**69.4**	**0**	**0.0**	**0**	**0.0**	**1**	**6.3**	**0**	**0.0**	**5**	**55.6**	**0**	**0.0**	**5**	**7.0**
Heterozygous deletion	42	18.5	5	8.5	5	71.4	0	0.0	22	61.1	0	0.0	0	0.0	1	6.3	0	0.0	4	44.4	0	0.0	5	7.0
Homozygous deletion	4	1.7	0	0.0	0	0.0	0	0.0	3	8.3	0	0.0	0	0.0	0	0.0	0	0.0	1	11.1	0	0.0	0	0.0
IKZF1	**46**	**20.2**	**6**	**10.2**	**6**	**85.7**	**0**	**0.0**	**3**	**8.3**	**0**	**0.0**	**0**	**0.0**	**11**	**68.8**	**0**	**0.0**	**1**	**11.1**	**0**	**0.0**	**19**	**26.8**
Heterozygous deletion	42	18.5	6	10.2	5	71.4	0	0.0	3	8.3	0	0.0	0	0.0	9	56.3	0	0.0	1	11.1	0	0.0	18	25.4
Homozygous deletion	4	1.7	0	0.0	1	14.3	0	0.0	0	0.0	0	0.0	0	0.0	2	12.5	0	0.0	0	0.0	0	0.0	1	1.4
IKZF1 **plus**	**22**	**9.9**	**2**	**3.4**	**4**	**57.1**	**0**	**0.0**	**1**	**2.8**	**0**	**0.0**	**0**	**0.0**	**4**	**25.0**	**0**	**0.0**	**1**	**11.1**	**0**	**0.0**	**10**	**14.1**
RB1	**29**	**12.4**	**3**	**5.1**	**6**	**85.7**	**1**	**25.0**	**2**	**5.6**	**0**	**0.0**	**2**	**14.3**	**3**	**18.8**	**0**	**0.0**	**1**	**11.1**	**0**	**0.0**	**11**	**15.5**
Heterozygous deletion	23	9.9	2	3.4	5	71.4	0	0.0	2	5.6	0	0.0	1	7.1	2	12.5	0	0.0	0	0.0	0	0.0	11	15.5
Homozygous deletion	5	2.1	1	1.7	1	14.3	1	25.0	0	0.0	0	0.0	0	0.0	1	6.3	0	0.0	1	11.1	0	0.0	0	0.0
Duplication	1	0.4	0	0.0	0	0.0	0	0.0	0	0.0	0	0.0	1	7.1	0	0.0	0	0.0	0	0.0	0	0.0	0	0.0
BTG1	**22**	**9.4**	**1**	**1.7**	**5**	**71.4**	**1**	**25.0**	**2**	**5.6**	**1**	**8.3**	**1**	**7.1**	**6**	**37.5**	**0**	**0.0**	**1**	**11.1**	**0**	**0.0**	**4**	**5.6**
Heterozygous deletion	19	8.2	0	0.0	5	71.4	1	25.0	2	5.6	1	8.3	0	0.0	5	31.3	0	0.0	1	11.1	0	0.0	4	5.6
Homozygous deletion	1	0.9	0	0.0	0	0.0	0	0.0	0	0.0	0	0.0	0	0.0	1	6.3	0	0.0	0	0.0	0	0.0	0	0.0
Duplication	2	0.4	1	1.7	0	0.0	0	0.0	0	0.0	0	0.0	1	7.1	0	0.0	0	0.0	0	0.0	0	0.0	0	0.0
EBF1 **heterozygous deletion**	**13**	**5.6**	**0**	**0.0**	**2**	**28.6**	**1**	**25.0**	**6**	**16.7**	**0**	**0.0**	**0**	**0.0**	**3**	**18.8**	**0**	**0.0**	**0**	**0.0**	**0**	**0.0**	**1**	**1.4**
ERG **heterozygous deletion**	**9**	**3.9**	**1**	**1.7**	**1**	**14.3**	**0**	**0.0**	**0**	**0.0**	**0**	**0.0**	**1**	**7.1**	**0**	**0.0**	**0**	**0.0**	**1**	**11.1**	**0**	**0.0**	**5**	**7.0**
**PAR1 deletion**	**2**	**0.9**	**1**	**1.7**	**0**	**0.0**	**0**	**0.0**	**0**	**0.0**	**0**	**0.0**	**0**	**0.0**	**1**	**6.3**	**0**	**0.0**	**0**	**0.0**	**0**	**0.0**	**0**	**0.0**

Supplementary Table S3. Detailed subtype, karyotype, MLPA P036 and DNA index results of B-ALL in this study.

ID	Subtype	Karyotype	MLPA P036	DNA index
778	Hyperdiploidy	64,XX,+X,+4,+5,+6,+8,+9,+11,+14,add(14)(q32),+15,+17,+18,+21,+22,+4mar[1]/63,XX,+X,+4,+5,+6,+8,+9,+11,+14,add(14)(q32),+15,+17,+18,+21,+22,+3mar[1]	61,XX,+X,+X,+4,+5,+6,+8,+9,+10,+11,+14,+14,+17,+18,+21,+21	NA
489	Hyperdiploidy	61,XY,+X,+4,+5,+6,+7,+8,+10,+12,+13,+14,+15,+17,+18,+21,+21[20]	55,XY,+5,+7,+8,+10,+11,+14,+18,+21,+21	1.183
990	Hyperdiploidy	56,XX,+X,+X,ins(1;?)(q21;?),+4,+6,del(9)(p?22),+10,+14,+17,+18,+21,+21[cp14]/46,XX[6]	52,XX,+X,+4,+6,+10,+17,+21	1.191
792	Hyperdiploidy	54,XX,+8,+14,+14,+17,+21,+2mar,inc[cp3]/46,XX[17]	51,XX,+6,+14,+17,+18,+21	1.189
938	Hyperdiploidy	46,XX[20]	52,XX,+X,+10,+14,+17,+18,+21	NA
341	Hyperdiploidy	46,XY[20]	58,XY,+X,+4,+5,+6,+8,+10,+11,+12,+14,+17,+18,+21	NA
800	Hyperdiploidy	46,XX[20]	55,XX,+X,+4,+6,+8,+10,+14,+17,+18,+21	1.221
889	Hyperdiploidy	53,XY,+X,+4,+6,+14,+17,+18,+21[15]/46,XY[5]	51,XY,+4,+6,+14,+18,+21	1.165
436	Hyperdiploidy	67,XX,+X,+3,+4,+5,+6,del(6)(q21,q25),+8,+9,+10,+11,+11,+12,+14,+14,+15,+16,+17,+18,+20,+21,+21,+mar[cp16]/46,XX[4]	NA	NA
159	Hyperdiploidy	46,XX[5]	53,XX,+5,+6,+10,+14,+17,+18,+21	1.150
899	Hyperdiploidy	46,XY[8]/56-60,XY,+X,+Y,tri(1)(1q),+4,+5,+6,del(6q),+7,+10,del(10q),+14,+17,+18,+20,+21,+21,+22,inc[cp12]	55,XY,+X,+5,+6,+10,+14,+18,+18,+21,+21	1.191
999	Hyperdiploidy	46,XY[4]	53,XY,+X,+4,+5,+6,+10,+14,+21	1.221
829	Hyperdiploidy/ *P2RY8-CRLF2*	46XY[20]	57,XY,+X,+X,+4,+8,+9,+10,+11,+14,+14,+21,+21	NA
706	Hyperdiploidy	58,XX,+4,der(4),t(1;4)(q?12;q?35),+5,del(5)(p13p15),+6,+8,+10,+11,+14,+17,+18,+21,+22,+X[13]/ 46,XX[7]	58,XX,+X,+4,+5,+6,+8,+10,+11,+14,+17,+18,+21,+21	NA
729	Hyperdiploidy	51,XY,+X,+4,-7,+10,+11,t(17;19)(q11.2;p13.3),+21,+21	51,XY,+X,+4,-7,+10,+11,+21,+21	NA

		[18]/46,XY[2]		
911	Hyperdiploidy	46,XY[20]	58,XY,+X,+4,+5,+6,+10,+12,+14,+16,+17,+18,+21,+22	1.218
442	Hyperdiploidy	46,XX[11]/62,XX,+X,+4,der(4)t(1;4)(q21;q28),+5,+6,+8,+9,+10,+11,i(12)(q10),+14,+15,+17,+18,+19,+21,+22,+22[10]	NA	NA
799	Hyperdiploidy	56,XY,+X,+4,+6,+8,+10,+14,+17,18,+21,+21[30]/57,idem,+9[3]/46,XY[2]	56,XY,+X,+4,+6,+8,+10,+14,+17,+18,+21,+21	1.217
919	Hyperdiploidy	54-57,XX,+X,+add(1)(p13),t(2;3)(q21;q21),+4,+6,add(6)(p25),del(6)(q23q25),+8,+14,+17,+18,+add(19)(q13),+21,del(22)(q11),inc[cp13]/46,XY[10]	54,XX,+4,+6,+8,+10,+14,+17,+18,+21	1.195
144	Hyperdiploidy	46,XX[6]	61,XX,+X,+1,+4,+5,+6,+9,+10,+16,+17,+18,+20,+20,+21,+21,+22	1.328
472	Hyperdiploidy	No mitosis	54,XY,+4,+6,+10,+14,+17,+18 +21,+21	NA
518	Hyperdiploidy	46,XY[20]	55,XY,+4,+6,+8,+10,+14,+17,+18,+21,+21	1.215
549	Hyperdiploidy	54-57,XY,+8,+10,+11,+15,+16,+21,inc[cp20]	55,XY,+X,+4,+8,+10,+11,+14,+18,+21,+21	NA
551	Hyperdiploidy	No mitosis	52,XX,+X,+4,+6,+14,+17,+21	NA
581	Hyperdiploidy	58-60,+XX,+X,+4,+5,del(6)(q15q21),+10,+11,del(12)(p11p13),+17,+19,+21,inc[cp9]/46,XX[1]	57,XX,+X,+4,+5,+10,+10,+11,+14,+17,+18,+21,+21	1.225
584	Hyperdiploidy	52,XX,+3,del(5)(q22,q31),+8,+9,+17,+18,+22[20]	54,XX,+X,+5,+6,+10,+14,+17,+21,+21	1.175
590	Hyperdiploidy	46,XX[2]	53,XX,+X,+6,+10,+14,+18,+21,+21	NA
686	Hyperdiploidy	65,XY,+X,+X,+3,+4,+5,+6,+8,+9,+10,+11,+11,+12,add(12)(p13),+14,+17,+18,+19,der(19)t(11;19)(q13;p13),+21,+21,+22[cp12]/46,XY[8]	63,XY,+X,+2,+3,+4,+5,+6,+7,+8,+9,+10,+11,+14,+17,+18,+21,+21,+22	NA
692	Hyperdiploidy	53,XY,+Y,+4,+6,+14,+17,+19,+21,+Mar[cp5]/46,XY[15]	54,XY,+4,+6,+10,+14,+17,+18,+21,+21	NA
698	Hyperdiploidy	No mitosis	54,XY,+X,+4,+6,+10,+14,+17,+18,+21	NA
712	Hyperdiploidy	48-51,XY,+X,+6,+8,+14,-16,+18,+21,+mar[cp20]	52,XY,+X,+6,+14,+18,+21,+21	1.110
720	Hyperdiploidy	54-57,XY,+X,+4,+6,+9,+10,+14,+17,+18,+19,+21,+21,	54,XY,+X,+4,+6,+10,+14,+17,+18,+21	1.162

		inc[cp16]/46,XY[4]		
737	Hyperdiploidy	46,XX[1]	54,XX,+X,+6,+10,+14,+14,+17,+18,+21	1.201
754	Hyperdiploidy	56,XX,+X,+3,+4,+6,+10,+10,+14,+18,+21,+21[13]/46,XX[7]	54,XX,+X,+3,+4,+10,+14,+18,+21,+21	1.225
761	Hyperdiploidy	46,XY[20]	54,XY,+X,+4,+6,+14,+17,+18,+21,+21	1.152
775	Hyperdiploidy	46,XX[20]	55,XX,+X,+X,+4,+10,+14,+17,+18,+21,+21	NA
794	Hyperdiploidy	47,XY,+21[5]/53-57,XY,+X,-Y,+5,+6,+15,+17,+21,+21,+1~8mar,inc[cp11]/46,XY[4]	55,XY,+X,+5,+6,+10,+15,+17,+18,+21,+21	1.181
833	Hyperdiploidy	46,XY[20]	53,XY,+X,+4,+6,+14,+17,+18,+21	1.160
836	Hyperdiploidy	46,XY[20]	54,XY,+X,+4,+6,+10,+14,+17,+18,+21	NA
840	Hyperdiploidy	46,XX[20]	53,XX,+X,+6,+10,+14,+17,+18,+21	1.134
846	Hyperdiploidy	46,XY[9]	54,XY,+X,+4,+7,+10,+14,+17,+18,+21	NA
852	Hyperdiploidy	63,XX,-3,add(3)(q13),+4,+5,+6,+6,del(6),+7,+8,+9,+10,+11,+12,+14,+14,+15,del(15)(q?12q?21),+16,+17,+20,+21,+21,inc[cp5]/46,XX[17]	57,XX,+4,+5,+6,+8,+14,+14,+17,+18,+21,+21,+22	NA
854	Hyperdiploidy	57,XY,+X,+4,+6,+7,+10,+14,+15,+17,+21,+21,+22[7]/46,XY[17]	56,XY,+4,+6,+8,+10,+14,+17,+18,+21,+21,+22	NA
870	Hyperdiploidy	53-55,XX,dup(1)(q21q32),+4,+6,+9,+12,+13,+14,+17,+18,+21,+22,inc[cp13]/46,XX[7]	54,XX,+X,+4,+6,+10,+14,+17,+18,+21	1.174
883	Hyperdiploidy	58,XX,+X,+dup1q,+4,+5,+6,+7,+8,+9,+10,+14,+14,+17,+18,+18,+21,+21,+marker[4]/46,XX[17]	58,XX,+X,+4,+6,+7,+8,+10,+14,+14,+17,+18,+21,+21	NA
885	Hyperdiploidy	58-59,XX,+X,+4,+6,+8,+10,+11+12,+14,+14,+19,+20,+21,+21,+22[18]/46,XX[2]	59,XX,+X,+4,+6,+8,+10,+11,+12,+14,+17,+18,+21,+21,+22	1.264
890	Hyperdiploidy	47,XY,-11,+21,+mar1,+mar2[1]/56,XY,+4,+12,+13,+17,-19,+21,+21,+22,+mar1~mar4[1]/46,XY[6]	55,XY,+X,+4,+6,+10,+14,+14,+15,+17,+21	NA
906	Hyperdiploidy	46,XX[3]/54-55,XX,+1,+1p,+4,+5,+6,+15,+17,+20,+21,+21,+22,inc[cp6]	53,XX,+4,+6,+14,+17,+18,+21,+21	1.126

917	Hyperdiploidy	56-58,XX,+X,+4,+6,+8,+9,+10,+11,+14,+17p,+18,+18,-20,+21,+21[17]/56-58,XX,+X,+4,+6,+8,+9,+10,+11,+14,+17p,+18,+18,-20,+21,+21,del(2p)[2]/46,XX[1]	55,XX,+X,+4,+6,+8,+10,+11,+14,+18,+21	1.226
921	Hyperdiploidy	46,XY[25]	55,XY,+X,+6,+9,+10,+14,+17,+18,+21,+21	NA
922	Hyperdiploidy	46,XY[25]	55,XY,+X,+4,+6,+9,+14,+17,+18,+21,+21	1.126
926	Hyperdiploidy	46,XY[11]/56-59,XY,+X,add(3q),+4,+5,+6,+8,+9,+10,+11,+12,add(12p),+14,+17,+18,+21,+21[cp9]	59,XY,+X,+4,+5,+6,+8,+10,+11,+12,+14,+17,+18,+21,+21	1.225
927	Hyperdiploidy	46,XY[20]	59,XY,+X,+4,+5,+6,+9,+10,+12,+14,+17,+18,+20,+21,+22	1.253
930	Hyperdiploidy	46,XX[17]	57,XX,+X,+4,+5,+6,+9,+10,+12,+14,+17,+18,+21	NA
960	Hyperdiploidy	46,XX[15]/54-55,XX,+X,add(1q)(q1),add(1q)(q21),+6,+8,+11,+17,+19,+20,+21,inc[cp5]	54,XX,+4,+6,+8,+10,+14,+17,+18,+21	1.199
964	Hyperdiploidy	46XY[1]	57,XY,+X,+4,+6,+8,+10,+14,+14,+17,+18,+21,+21	1.209
968	Hyperdiploidy	54,XY,+4,+6,+8,+10,+14,+18,+21,+21[1]/46,XY[3]	54,XY,+X,+4,+5,+10,+14,+17,+21,+21	1.176
973	Hyperdiploidy	46,XX[4]	57,XX,+X,+4,+8,+10,+11,+14,+15,+17,+18,+21,+21	1.263
977	Hyperdiploidy	54,XY,+X,+6,+10,+14,+17,+18,+21,+21[15]/46,XY[5]	53,XY,+X,+6,+10,+14,+17,+18,+21	1.138
753	Masked Hypodiploidy	52-54,XX,+mar1~mar8[cp4]/46,XX[21]	27,X,-1,-2,-3,-5,-6,-7,-9,-10,-11,-12,-13,-15,-16,-17,-18,-19,-20,-22	1.17
952	Hypodiploidy	26,XY,-1,-2,-3,-4,-5,-6,-7,-8,-9,-11,-12,-13,-14,-15,-16,-17,-18,-19,-20,-22[13]	26,XY,-1,-2,-3,-4,-5,-6,-7,-8,-9,-11,-12,-13,-14,-15,-16,-17,-18,-19,-20,-22	0.561
508	Hypodiploidy	46,XY[20]	32,XY,-2,-3,-4,-6,-7,-9,-10,-12,-15,-16,-17,-18,-20,-22	NA
984	Masked Hypodiploidy	63,XX,-X,+1,-3,add(3)(q13),del(3)(q?21),-4,-5,+6,-7,-9,-9,-10,+12,-13,+14,-15,-16,-17,-20,+add(21)(q22)x2,+22,+2mar,inc[cp14]/46,XX[6]	33,XX,-3,-4,-5,-7,-8,-9,-10,-11,-13,-15,-16,-17,-20	0.789/ 1.535
925	Hypodiploidy	42,X,-4,-9,-13,t(14;17)(q32;p11.2),add(21)(p11.2)[16]/84,idem x2[3]/46,XY[1]	42,Y,-4,-9,-13	0.914
845	Masked Hypodiploidy	68,XX,-Y,+1,-2,-3,-4,del(4)(q21q31),del(5)(q13q33),+6,-7,+8,+9,-10,+11,-12,-13,+14,-15,-16,-17,-18,+19,+20,	34,Y,-2,-3,-4,-7,-10,-12,-13,-15,-16,-17-18	0.76/ 1.43

		+21,+22,inc[cp5]/46,XY[20]		
774	Hypodiploidy	46,XY[25]	38,XY,-2,-3,-12,-13,-14,-15,-16,-17	0.818
689	iAMP21	46,XX[20]	46,XX,+10,-20	1
813	iAMP21	46,XX[20]	48,XX,+X,+10	NA
306	iAMP21	46,XY[14]/45,XY,-20q,-21[6]	45,XY,-21	NA
188	iAMP21	46,XX,-21[12]/46,XX[2]	46,XX	1
545	*ETV6-RUNX1*	45,X,t(5;12)(q13;p13)[18]/46,XX[2]	45,X	NA
857	*ETV6-RUNX1*	44,XX,-7,+9p,+11q,-15,+15p,+16q,+21p,+mar[7]/ 46,XX[13]	46,XX	NA
982	*ETV6-RUNX1*	46,XX,del(8)(q12q13),del(11)(q21q23),del(12)(p12), dup(17)(q21q25)[cp11]/46,XX[9]	46,XX	1
631	*ETV6-RUNX1*	No mitosis	46,XY	NA
745	*ETV6-RUNX1*	47,XY,+21[3]/47,idem,del(2)(q35)[1]/49,idem,+6,+19 [1]/46,XY[4]	47,XY,+21	NA
789	*ETV6-RUNX1*	NA	47,XX,+10	NA
933	*ETV6-RUNX1*	46,XX,del(6)(q13)[14]/46,XX[6]	46,XX	1
963	*ETV6-RUNX1*	46,XY,t(5;12)(q12;p12),del(11)(q14)[10]/45-46,XY, t(1;8)(q23;q21.1),t(5;12)(q12;p12),del(11)(q14)[cp8]/ 46,XY[1]	46,XY	NA
724	*ETV6-RUNX1*	46,XY[8]	46,XY,-13,+21	NA
894	*ETV6-RUNX1*	45,X,-X,add(2)(p11.2),-8,+mar[1]/47,XX,+mar[1]/ 46,XX[3]	46,XX	NA
902	*ETV6-RUNX1*	No mitosis	46,XY	NA
949	*ETV6-RUNX1*	46,XY[20]/46,XY,del(11)(q23)[1]	46,XY	1
986	*ETV6-RUNX1*	47,XY,dic(12;19)(p11;p11),t(18;19)(q11;p13),+19[20]	47,XY,+21	1
989	*ETV6-RUNX1*	46,XY,t(8;12)(p10;p10)[10]/46,XY[10]	46,XY	1
796	*ETV6-RUNX1*	46,XX,del(12)(p11p13)[2]/46,XX[1]	NA	NA
713	*ETV6-RUNX1*	No mitosis	46,XY	NA

546	*ETV6-RUNX1*	46,XX[20]	46,XX	NA
463	*ETV6-RUNX1*	46,XY[15]	NA	NA
347	*ETV6-RUNX1*	46,XY[14]	46,XY	NA
553	*ETV6-RUNX1*	No mitosis	46,XY	NA
614	*ETV6-RUNX1*	47,XY,+10,del(12)(p11p13)[1]/46,XY[4]	47,XY,+10	NA
888	*ETV6-RUNX1*	48,XX,+10,t(12;19)(p11;p13),+21[17]/46,XX[3]	48,XX,+10,+21	NA
510	*ETV6-RUNX1*	46,XY[20]	NA	NA
886	*ETV6-RUNX1*	46,XX,add(6)(q?15),del(12)(p11p13),+21[7]/46,XX, idem,add(16)(q22)[13]	46,XX	NA
940	*ETV6-RUNX1*	46,XX[3]/46-47,X,add(12p),add(18p),+mar,+mar[cp7]	46,XX	1
676	*ETV6-RUNX1*	46,XX[6]	46,XX	NA
747	*ETV6-RUNX1*	46,XX[8]	NA	NA
752	*ETV6-RUNX1*	46,XY[20]	47,XY,+21	NA
806	*ETV6-RUNX1*	46,XY[20]	46,XY	NA
844	*ETV6-RUNX1*	No mitosis	46,XY	NA
877	*ETV6-RUNX1*	46,XX[20]	46,XX	NA
974	*ETV6-RUNX1*	No mitosis	46,XX	NA
995	*ETV6-RUNX1*	46,XX[20]	46,XX	1.069
351	*ETV6-RUNX1*	46,XX[20]	45,X	NA
767	*ETV6-RUNX1*	46,XY[20]	46,XY	NA
682	*ETV6-RUNX1*	46,XY[5]	46,XY	NA
386	*TCF3-PBX1*	46,XY,t(1;19)(q23;p13)[20]	46,XY	NA
868	*TCF3-PBX1*	46,XY,der(19)t(1;19)(q23;p13)[11]/46,XY[9]	46,XY	NA
918	*TCF3-PBX1*	46,XX[5]	46,XX	1
515	*TCF3-PBX1*	42-47,XY,t(1;19)(q23;p13)[cp12]	45,XY,-17	NA
912	*TCF3-PBX1*	46,XX,rea(9p),add(15)(p12),der(19)t(1;19)(q23;p13.3), -21,+22[cp15]/46,XX[5]	46,XX	1
612	*TCF3-PBX1*	48,XY,t(1;19)(q23;p13),dup(6)(p21p25),+8,+20[19]/	48,XY,+8,+20	NA

		46,XY[1]		
679	*TCF3-PBX1*	46,XX[20/20]	46,XY	NA
688	*TCF3-PBX1*	46,XX,der(19),t(1;19)(q23;p13)[7]/46,XX, t(1;19)(q23;p13)[5]/46,XX[8]	46,XX	NA
700	*TCF3-PBX1*	46,XX,der(19)t(1:19)(q23;p13)[9]/46,idm, add(3)(q21)[2]	46,XX	NA
728	*TCF3-PBX1*	46,XX,t(1;19)(q23;p13)[10]/46,XX,del(6)(q21q25), del(11)(q23),der(19),t(1;19)[4]/88,XXXX,-4,-17,-18, -20[1]/46,XX[5]	46,XX	NA
898	*TCF3-PBX1*	46,XX[20]	46,XX	NA
976	*TCF3-PBX1*	46,XY,t(1;19)(q23;p13)[13]/46,XY[7]	46,XY	1
658	*KMT2A-EPS15*	46,XX,t(1;11)(p32;q23)[20]	46,XX	NA
965	*KMT2A-AFF1*	No mitosis	46,XX	1.055
657	*KMT2A-MLLT3*	46,XY,t(9;20)[1]/46,XY[1]	45,XY,-17,-19,+22	1
331	*KMT2A-AFF1*	46,XY,rea(9)(p13),rea(11)(q23)[20]	NA	NA
471	*KMT2A-EPS15*	46,XX,t(1;11)(p32;q23)[4]/46,XX[6]	46,XX	NA
953	*KMT2A-MLLT1*	50,XY,+X,+6,+21,+22,t(11;19)[20]	50,XY,+X,+6,+21,+22	1
373	*KMT2A-MLLT1*	48,XX,+X,+6,t(11;19)(q23;p13)[5]	48,XX,+X,+6	NA
554	*KMT2A-AFF1*	46,XY,t(4;11)[20]	46,XY	NA
623	*KMT2A-AFF1*	46,XX,t(4;11)(q21;q23)[14]/46,XX[7]	46,XX	1
630	*KMT2A-AFF1*	46,XY,t(4;11)[10]/46,XY[10]	46,XY	NA
649	*KMT2A-AFF1*	50,XY,+X,+1,+4,t(4;11)(q21;q23),+8,i(17)(q10)[6]/ 50,XY,idem,+21[14]	50,XY,+1,+4,+8,+20	NA
656	*KMT2A-AFF1*	46,XY,t(4;11)(q21;q23)[16]/47,idem,+8[4]	46,XY	NA
748	*KMT2A-AFF1*	46,XX,t(4;11)(q21;q23)[13]/47,idem,+X[1]/48,XX,idem ,+X,+22[1]/47,XX,idem,+X,-5,-5,+del(6)(q21;q23)x2 [1]/46,XX[2]	NA	NA
864	*KMT2A-MLLT10*	46,XX[14]/t(10;11)(p13q21)[6]	46,XX	1

437	*BCR-ABL1*	46,XX,t(9;22)(q34;q11.2)[20]	NA	NA
798	*BCR-ABL1*	46,XY,del(3)(q12q29),del(7)(p13p15),i(8)(q10), t(9;22)(q34;q11.2)[3]/46,XY[3]	46,XY	NA
598	*BCR-ABL1*	46,XY,t(8;14)(p21;q32)[6]/46,s1,t(9;22)(q34;q11.2), add(17)(q25)[14]	46,XY	NA
705	*P2RY8-CRLF2*	46,XY,t(8;9)(q11;p13)[22]	46,XY	NA
910	*BCR-ABL1*	46,XX[25]	46,XX	NA
362	*EPOR-IGH*	46,XY,t(9;10)(p22;q24)[9]/47-49,idem,+8,+21,+21,+22 [cp3]/45,idem,del(4)(p15p16),-7,der(10)t(9;10), add(15)(p11),+mar[3]/46,XY[5]	46,XY	NA
937	*BCR-ABL1*	46,XX[4]/46,XX,t(9;22)[13]/47,XX,der(22),t(9;22)[3]	46,XX	1
855	*BCR-ABL1*	46,XX,t(6;12),t(9;22)(q34;q11.2)[20]	NA	NA
997	*EBF1-PDGFRB*	46,XY[6]	46,XY	1
520	*BCR-ABL1*	45,XY,der(9)t(9;22)(q34;q11.2),add(19)(p13),-22[15]/ 45,idem,del(11)(q23)[1]	NA	NA
469	*BCR-ABL1*	46,XX,t(9;22)(q34;q11.2)[17]	NA	NA
456	*BCR-ABL1*	46,XY,t(9;22)(q34;q11.2)[11]/47,idem,+der(22)t(9;22) [10]/48,idem,+8,+der(22)t(9;22)[3]/46,XY[1]	NA	NA
171	*EBF1-PDGFRB*	46,XY[3]	46,XY	1
892	*BCR-ABL1*	46,XY[3]	46,XY	NA
435	*BCR-ABL1*	47,XY,del(1q),+3,del(9q)[20]	51,XY,+X,+2,+6,+14,+21	NA
667	*BCR-ABL1*	46,XY,t(9;22)(q34;q11.2)[20]	46,XY	NA
439	*TCF3-HLF*	45,XY,del(6)(q13,q25),der(9),add(9)(p24), del(9)(q13q22),der(9),t(9;9)(p12;q21),-14, t(17;19)(q22;p13)[11]/46,XY[9]	46,XY	NA
784	*TCF3-HLF*	46,XY,t(17;19)(q22;p13)[12]/46,XY,der(8),t(8;8), t(17;19)(q22;p13),add(19p)[12]/49,XY,add(1p),+6,+7, add(19p),-20,+22,+mar[1]	46,XY	1

955	*AKAP8-ZNF384*	46,XX[20]	46,XX	1
929	*EWSR1-ZNF362*	46,XY,add(1)(p32),add(12)(q24.1),-13,-22,+2mar[3]/ 48,idem,add(1)(p32),+8[15]/56,idem,+X,add(1)(p32), +4,+8,+11,+11,+14,+17,+18,+19,+mar[8]/46,XY[2]	46,XY	1
703	*EP300-ZNF384*	NA	46,XX	1
1003	*TCF4-ZNF384*	58,XY,+X,+1,+3,+5,+6,+7,+8,+9,+11,+15,+17,+22[1]/ 46,XY[19]	46,XY	1
131	*TCF3-ZNF384*	46,XX[8]	46,XX	1
947	*EP300-ZNF384*	46,XX[9]/45,X,t(11;12),+22q,inc[11]	45,X	1
434	*TCF3-ZNF384*	47,XX,+8[2]/46,XX[18]	47,XX,+8	NA
983	*EP300-ZNF384*	46,XY[11]	46,XY	1
772	*TCF3-ZNF384*	46,XX,+4p,+16,-19[1]/46,XX[5]	46,XX	1
970	*MEF2D-HNRNPUL1*	46,XX,del(9p)[20]	46,XX	1
832	*MEF2D-BCL9*	46,XX,del(3)(q23)[1]/46,XX,-13,+mar[1]/45,XX,-13, -14,+mar[1]	46,XX	NA
507	*MEF2D-BCL9*	46,XX[20]	46,XX	NA
576	Other	46,XY[20]	46,XY	NA
954	Other	46,XX,del(9)(p13)[1]/46,XX[24]	46,XX	1
783	Other	46,XX[20]	47,XX,+8,-9,+18	NA
985	Other	46,XY[25]	45,XY,-7	1
543	Other	46,XY[1]	45,XY,-7	1
909	Other	46,XY[9]	44,XY,-7,-9	NA
299	Other	46,XY,t(1,7)[20]	46,XY	1
779	Other	No mitosis	47,XY,+5	1
914	Other	46,XY,del(9)(p22)[21]	46,XY	1
710	Other	46,XX[16]	45,XX,-7	1
683	Other	46,XY[4]	46,XY	1
503	Other	47,XY,+X,rea(20p)[2]/46,XY[18]	47,XY,+X	1

872	Other	45,XX,-20[14]/46,XX[6]	46,XX	1
193	Other	NA	49,XY,+X,+4,+12	1.085
856	Other	45,XY,der(9),t(9;16)(p13;q13),-16[9]/46,XY[14]	NA	1
476	Other	45,XY,t(1;8)(p34;p24),rea(11q),-18[16]/44,XY,iden,-21[3]/46,XY[2]	46,XY	1
628	Other	47,XY,+9[19]/47,idem,+18,-21[1]	47,XY,+9	1
777	Other	46,XX,t(2;5)(p13;q13),del(11)(q21q23)[5]/46,idem, del(7)(p13p15)[8]/46,XX[7]	46,XX	1
441	Other	46,XY,t(9;19)[20]	NA	NA
473	Other	46,XX[20]	46,XX	NA
646	Other	No mitosis	45,X	1
699	Other	47,XX,+21[1]	47,XX,+21	1
781	Other	NA	48,XY,+8,+18	1
863	Other	47,XX,der(1;9)(q10;q10),add(17)(p13),+mar[6]/46,XX[19]	46,XX	1
979	Other	46,XX,t(14;19)(q32;q13)[4]/47,idem,+4[15]/46,XX[1]	47,XX,+4	1.055
495	Other	No mitosis	NA	NA
690	Other	46,XY[20]	46,XY	1
814	Other	46,XX[20]	46,XX	1
537	Other	45,XX,-4p,-9q,-18,+20q[18]/46,XX[2]	NA	NA
338	Other	46,XY[20]	NA	NA
632	Other	45-46,XX,del(11)(q13,q23),add(12)(p13),inc[cp4]/ 46,xx[3]	46,XX	1
563	Other	50,XX,+X,+17,+21,+21[13]/46,XX[9]	50,XX,+X,+17,+21,+21	1.075
452	Other	NA	NA	NA
282	Other	45,X,-Y[5]/46,XY[11]	46,XY	1
470	Other	46,XX[10]	NA	NA
787	Other	46,XX[20]	46,XX	1

807	Other	46,XY[4]	46,XY	1
450	Other	NA	NA	NA
438	Other	46,XY[19]/46,XY,-8q[1]	46,XY	NA
696	Other	46,XY[20]	46,XY	NA
782	Other	45,X,-Y[1]/46,XY[19]	46,XY	1
164	Other	46,X,del(Y)(q1?2),t(5;7)(p13;p11),add(7)(p14)[5]/46, XY,t(13;14)(q12;q32)[1]/46,XY[1]	47,XY,+20	1
50	Other	46,XX[20]	45,X,-9	1
804	Other	46,XY[20]	46,XY	1
866	Other	46,XY[20]	46,XY	1
981	Other	46,XX,t(3;11)(p21;q23)[cp17]/46,XX[3]	46,XX	NA
337	Other	46,XY[20]	NA	NA
404	Other	46,XY[20]	NA	NA
448	Other	46,XX[5]	NA	NA
461	Other	46,XX,rea(1q),rea(19q)[cp9]/46,XX[11]	NA	NA
462	Other	46,XY[20]	NA	NA
460	Other	52,XY,+X,+Y,+9,+9,-17,+21,+21,+mar[8]/46,XY[2]	NA	NA
564	Other	42,Y,-X,-2,-7,-22[1]/40,XY,-5,-8,-9,-11,-13,-13[2]	46,XY	NA
372	Other	46,XY[20]	46,XY	NA
544	Other	46,XX[8]	46,XX	1
893	Other	46,XX[24]	46,XX	NA
474	Other	46,XY,t(2;16)(p10;q10),del(17)(q11.2)[4]/47,idem,+mar [1]/46,XY,del(3)(p10),del(11p)[1]/46,XY[14]	NA	NA
425	Other	46,XX,i(17)(q10)[4]/46,XX[12]	46,XX	NA
529	Other	46,XY[20]	46,XY	NA
541	Other	46,XX[20]	46,XX	1
653	Other	46,XY[7]	46,XY	1
734	Other	46,XY,t(7;15)(q22;q15)[8]/46,XY[12]	46,XY	1

738	Other	46,XY,del(6)(q?13q?23)[1]/46,XY[13]	46,XY	1
776	Other	46,XX[22]	46,XX	NA
786	Other	46,XX,t(8;14)[20]	46,XX	NA
790	Other	46-47,XY,i(5)(q10),del(9)(p21p24),del(12)(p11),+2mar,inc[cp2]/46,XY[3]	47,XY,+18	1
827	Other	48,XY,+5,+21[12]/46,XY[8]	48,XY,+5,+21	1
928	Other	46,XX[17]	47,XX,+21	1
959	Other	46,XX[3]	46,XX	1
465	Other	46,XX[7]	NA	NA
440	Other	46,XY[20]	46,XY	NA

Supplementary Table S4. STR profiling of masked hypodiploidy and high hyperdiploidy B-ALL cases.

Patient ID		Pt984					Pt845				Pt753*			Pt990			
Ploidy Group		Masked Hypodiploidy					Masked Hypodiploidy				Masked Hypodiploidy			High Hyperdiploidy			
		Origin		STR loss in tumor	CN	LOH	Origin		STR loss in tumor	CN	Origin	CN	LOH	Origin		STR loss in tumor	CN
STR loci	Chr.	Tumor	Germline				Tumor	Germline			Tumor			Tumor	Germline		
D1S1656	1	13/16.3	13/16.3	N	Gain	Normal	12/15	12/15	N	Gain	14/14	Normal	LOH	11/14	11/14	N	Normal
D2S1338	2	20/24	20/24	N	Gain	Normal	25/25	19/25	Y	Normal	19/19	Normal	LOH	19/23	19/23	N	Normal
D2S441	2	11/12	11/12	N	Gain	Normal	12/12	10/12	Y	Normal	12/12	Normal	LOH	11/12	11/12	N	Normal
TPOX	2	8/12	8/12	N	Gain	Normal	9/9	9/11	Y	Normal	8/8	Normal	LOH	8/11	8/11	N	Normal
D3S1358	3	15/15	15/15	N	Normal	LOH	16/16	16/16	N	Normal	17/17	Normal	LOH	16/17	16/17	N	Normal
FGA	4	20/20	20/21	Y	Normal	LOH	25/25	23/25	Y	Normal	19/24	Gain	Normal	21/22	21/22	N	Gain
CSF1PO	5	12/12	12/12	N	Gain	LOH	10/12	10/12	N	Gain	12/12	Normal	LOH	8/10	8/10	N	Normal
D5S818	5	11/11	11/12	Y	Normal	LOH	11/13	11/13	N	Gain	10/10	Normal	LOH	9/11	9/11	N	Normal
D6S1043	6	19/19	19/19	N	Gain	Normal	19/20	19/20	N	Gain	18/18	Normal	LOH	11/13	11/13	N	Gain
D7S820	7	8/8	8/11	Y	Normal	LOH	12/12	11/12	Y	Normal	12/12	Normal	LOH	10/12	10/12	N	Normal
D8S1179	8	17/17	13/17	Y	Normal	LOH	10/13	10/13	N	Gain	10/13	Gain	Normal	14/15	14/15	N	Normal
D10S1248	10	16/16	15/16	Y	Normal	LOH#	13/13	12/13	Y	Normal	13/13	Normal	LOH	13/15	13/15	N	Gain
TH01	11	10/10	10/10	N	Gain	Normal	7/9.3	7/9.3	N	Gain	9/9	Normal	LOH	6/8	6/8	N	Normal
vWA	12	14/18	14/18	N	Gain	Normal	15/15	15/18	Y	Normal	16/16	Normal	LOH	17/17	17/17	N	Normal
D12S391	12	16/21	16/21	N	Gain	Normal	17/17	17/20	N	Gain	18/19	Normal	LOH	18/21	18/21	N	Normal
D13S317	13	11/11	9/11	Y	Normal	LOH	11/11	11/11	N	Normal	11/11	Normal	LOH	8/9	8/9	N	Normal
D16S539	16	11/11	11/11	N	Normal	LOH	9/9	9/11	Y	Normal	9/9	Normal	LOH	10/11	10/11	N	Normal
D18S51	18	18/21	18/21	N	Gain	Normal	19/19	13/19	Y	Normal	16/16	Normal	LOH	13/15	13/15	N	Normal
D19S433	19	15.2/16.2	15.2/16.2	N	Gain	Normal	14/14	14/14	N	Gain	14/14	Normal	LOH	13/14.2	13/14.2	N	Normal
D21S11	21	28/32.2	28/32.2	N	Gain	Normal	29/32.2	29/32.2	N	Gain	28/30	Gain	Normal	30/31	30/31	N	Gain
D22S1045	22	11/15	11/15	N	Gain	Normal	15/17	15/17	N	Gain	11/11	Normal	LOH	11/17	11/17	N	Normal
Amelogenin	X/Y	X/X	X/X	N	Gain	Normal	X/X	X/Y	Y	Normal	X/X	Normal	LOH	X/X	X/X	N	Normal

STR: short tandem repeat; Y: Yes; N: No; CN: copy number; LOH: loss of heterozygosity

*: germline sample was not available for testing; # LOH on 10q only

LOH was interpreted using CytoScan array result; CN was interpreted using either CytoScan array or MLPA P036

Supplementary Table S5. 5-year EFS and OS using univariate and multivariate survival analysis.

5-year EFS						
	Univariate			Multivariate		
	HR	95% CI	P-value	HR	95% CI	P-value
IKZF1	1.61	0.94-2.77	0.083	1.07	0.60-1.91	0.820
Age	2.09	1.26-3.48	0.005	2.15	1.26-3.68	0.005
Gender	0.55	0.33-0.93	0.024	0.57	0.34-0.97	0.039
WBC	2.06	1.16-3.64	0.013	1.89	1.06-3.38	0.032
Protocol	0.38	0.20-0.73	0.004	0.36	0.18-0.70	0.003
5-year OS						
	Univariate			Multivariate		
	HR	95% CI	P-value	HR	95% CI	P-value
IKZF1	1.07	0.54-2.10	0.854	0.62	0.30-1.29	0.204
Age	2.27	1.27-4.07	0.006	2.78	1.51-5.14	0.001
Gender	0.51	0.28-0.95	0.034	0.50	0.27-0.94	0.032
WBC	2.47	1.32-4.64	0.005	2.45	1.28-4.69	0.007
Protocol	0.37	0.16-0.86	0.020	0.40	0.18-0.93	0.033

EFS, event-free survival; OS, overall survival; HR, hazard ratio; CI, confidence interval.

IKZF1 (reference = *IKZF1* wild type); Age (reference = <10 years); Gender (reference = female); WBC (reference = <100 x 10^9/liter); Protocol (reference = TPOG-2002).

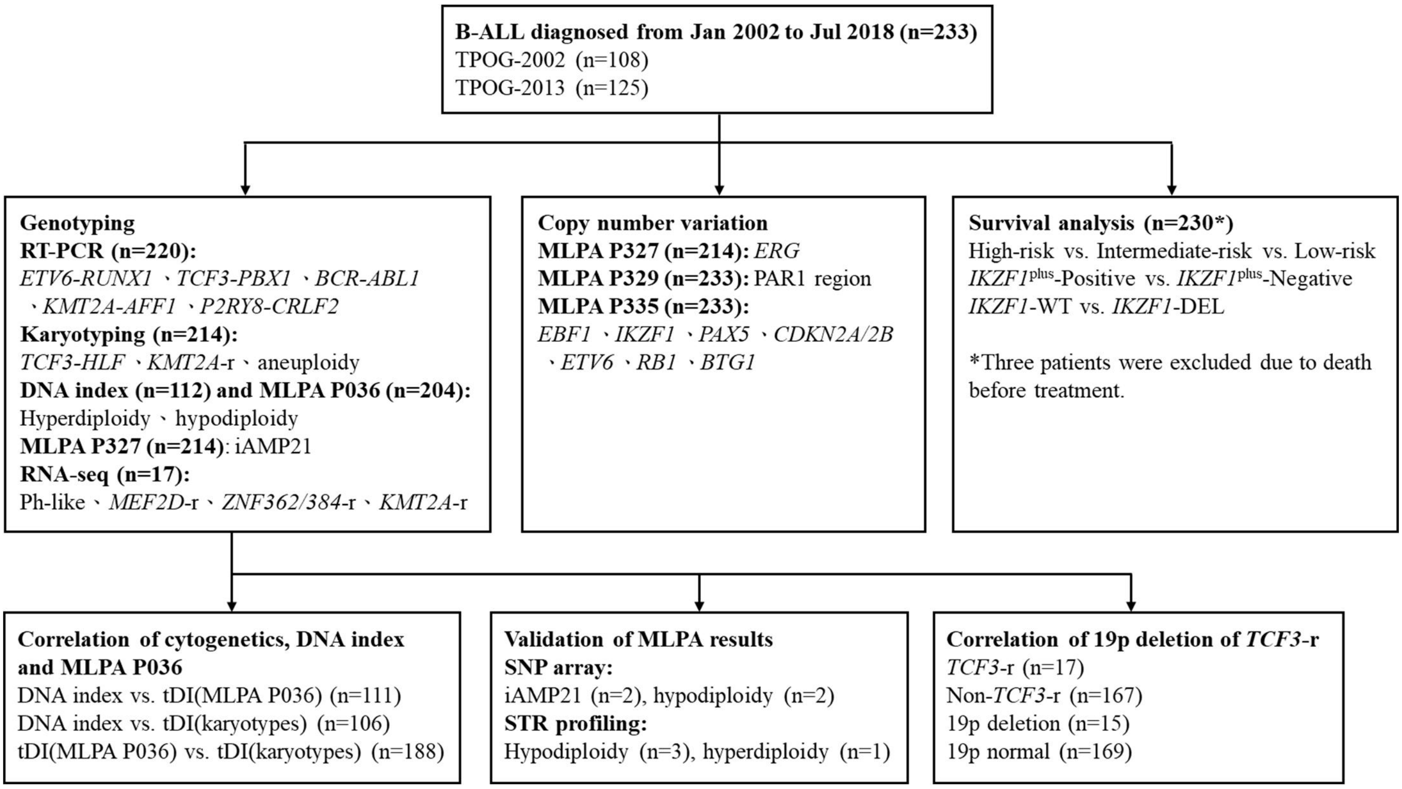

Supplementary Figure S1. Flow diagram of analysis through this study.

Types of analysis and patient numbers assayed in each analysis were demonstrated.

(a) (b)

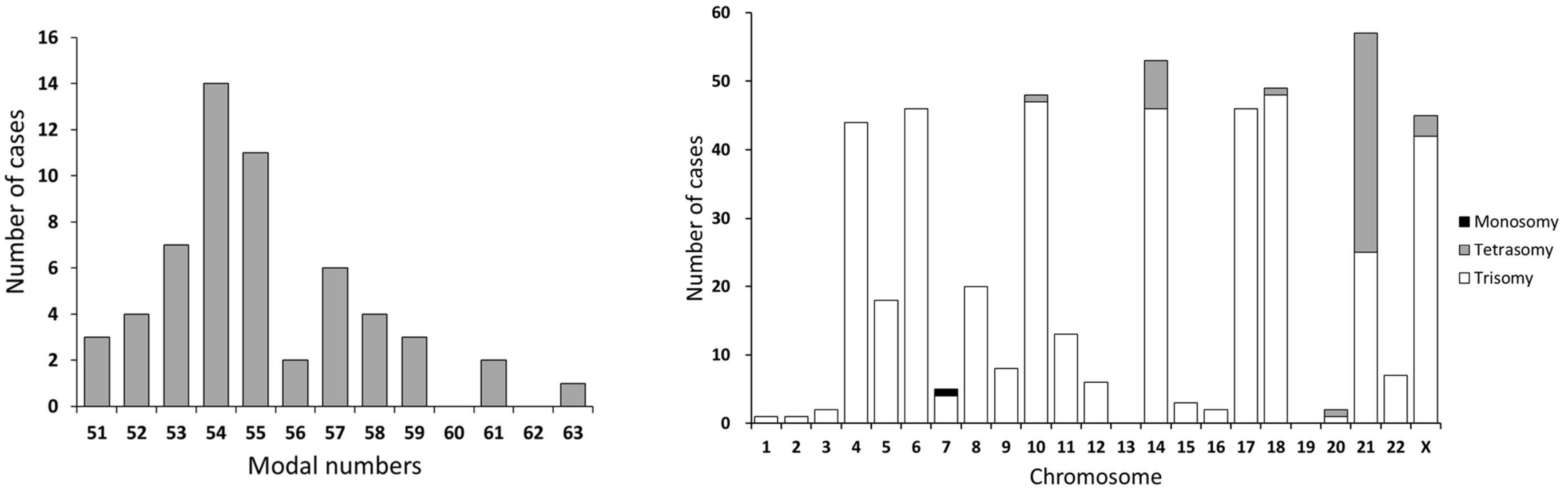

Supplementary Figure S2. Analysis of high hyperdiploidy cases.

(a) Distribution of 57 cases of childhood hyperdiploid acute lymphoblastic leukemia cases by modal chromosome number. (b) Number of childhood hyperdiploid acute lymphoblastic leukemia cases with monosomy, trisomy, and tetrasomy of each chromosome (N =57).

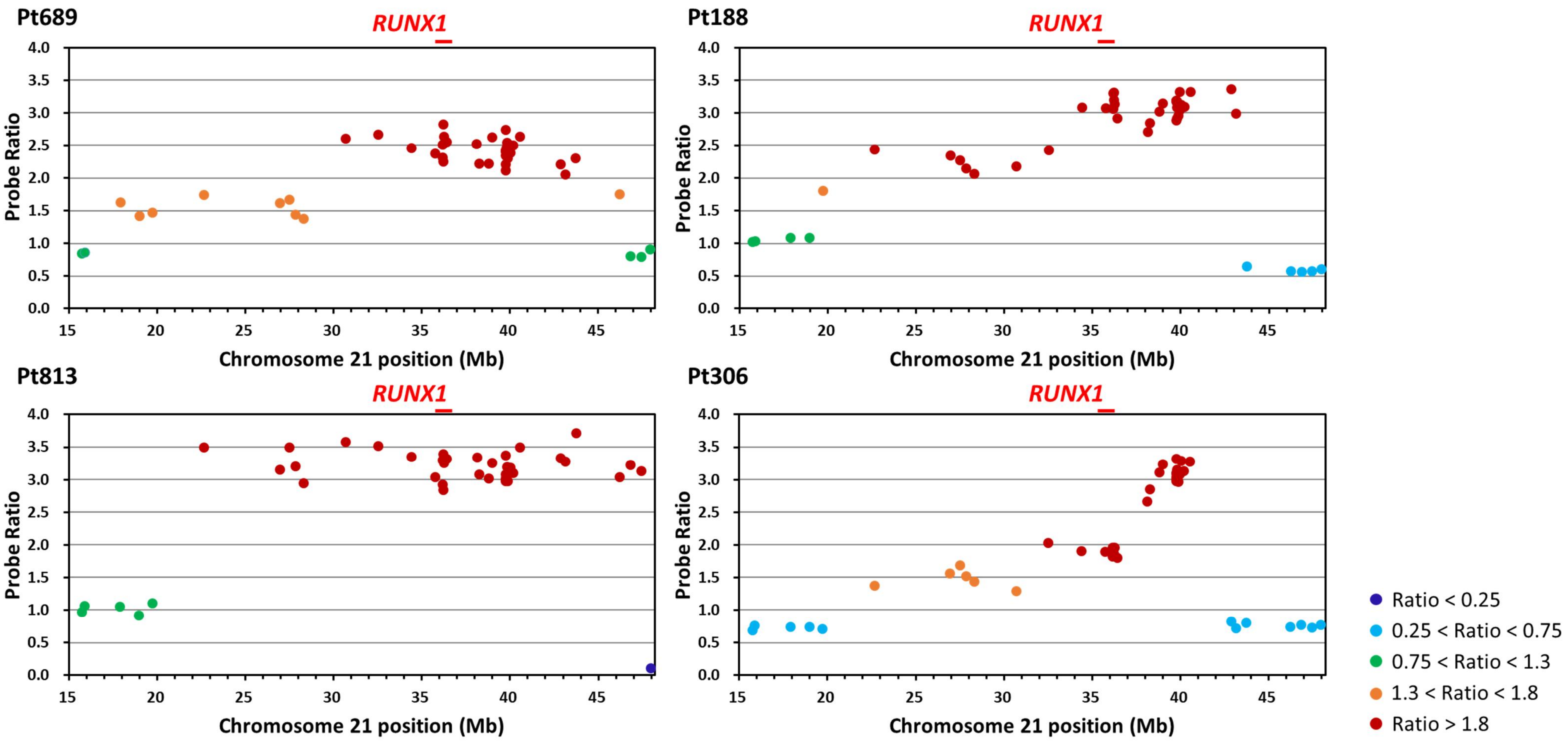

Supplementary Figure S3. The MLPA P327 of iAMP21-ALL.

The *RUNX1* (36.16-36.42 Mb) is the highest level of amplification on chromosome 21 in three cases of iAMP21 in this cohort. However, *RUNX1* is not within the highest level of amplification on chromosome 21 in patient 306.

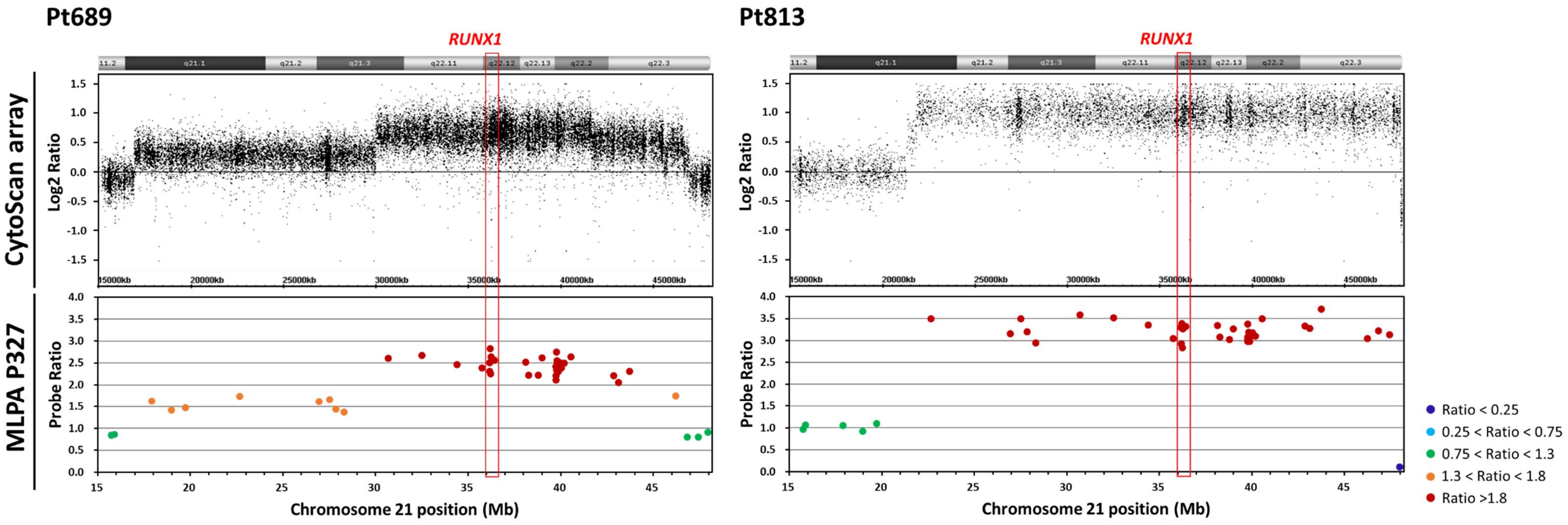

Supplementary Figure S4. The results of CytoScan HD (Pt689) or 750K (Pt813) array.

The results of array are compatible with the results of MLPA P327 and both of methods are able to detect iAMP21. The genomic location of *RUNX1* (36.16-36.42 Mb) is highlighted.

(a)

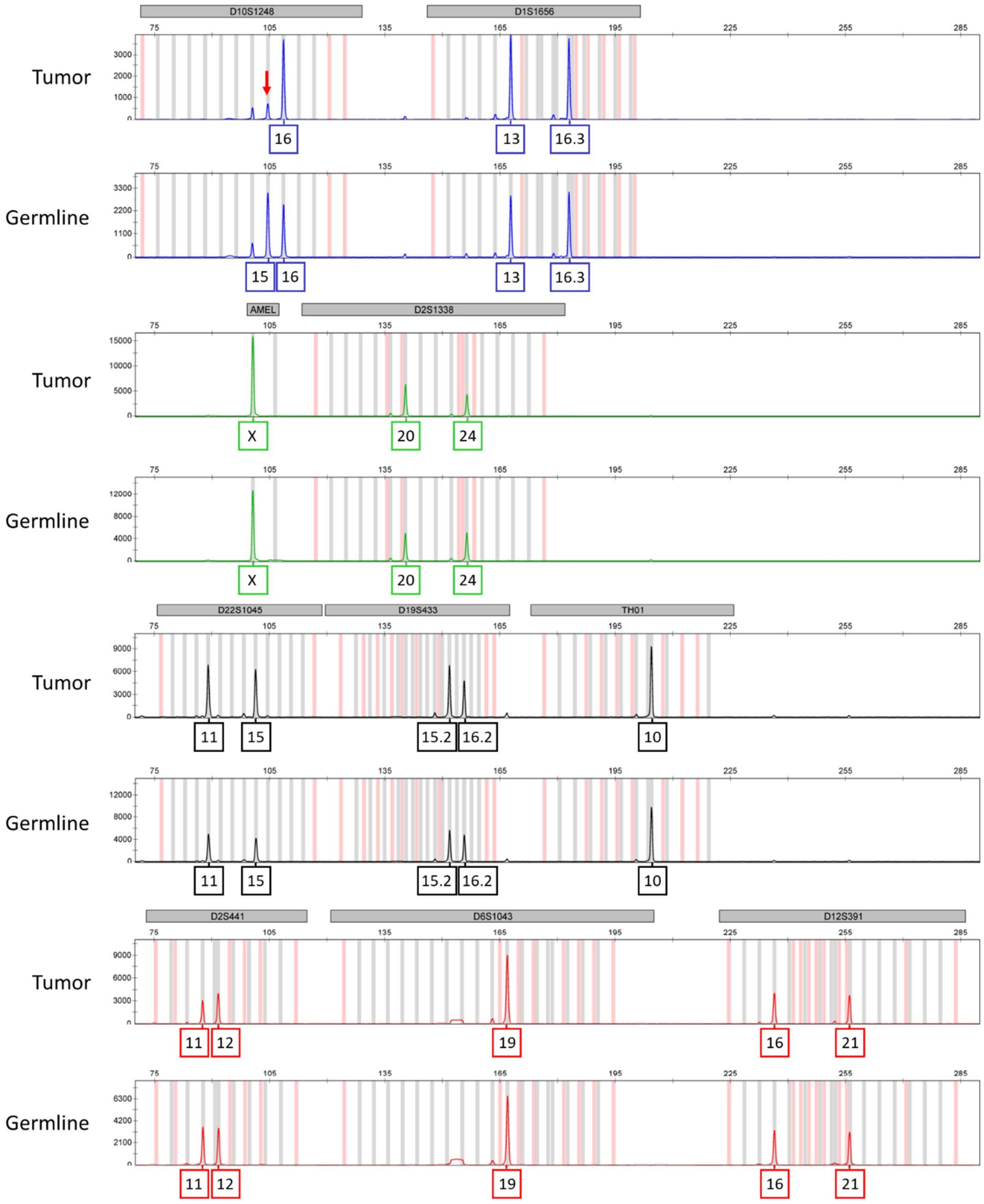
D10S1248
D1S1656
Tumor
16
13
16.3
Germline
15
16
13
16.3
AMEL
D2S1338
Tumor
X
20
24
Germline
X
20
24
D22S1045
D19S433
TH01
Tumor
11
15
15.2
16.2
10
Germline
11
15
15.2
16.2
10
D2S441
D6S1043
D12S391
Tumor
11
12
19
16
21
Germline
11
12
19
16
21

(b)

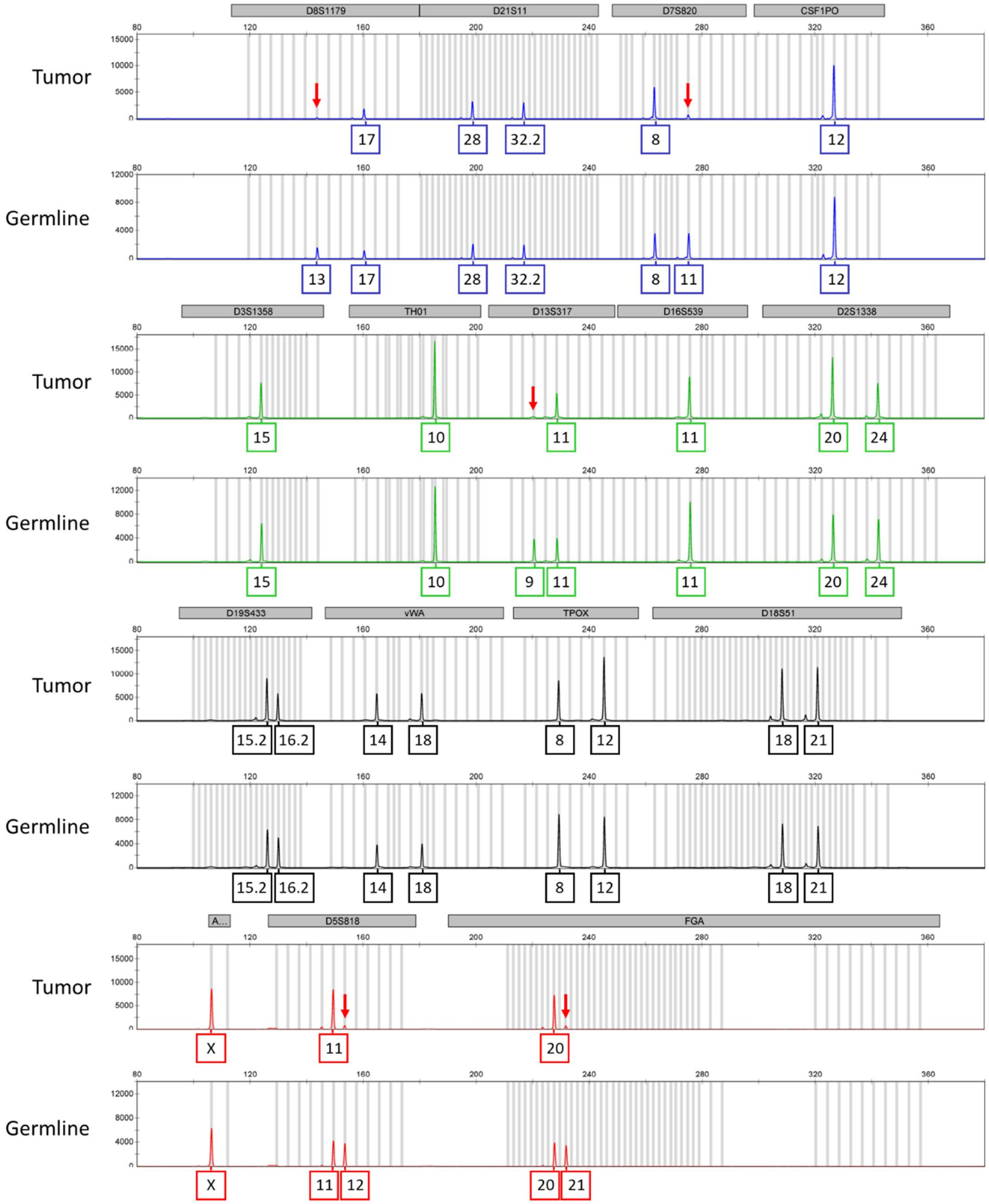

Supplementary Figure S5. STR profiling for masked hypodiploidy.

(a) VeriFiler™ and (b) Identifier™ Direct kit for tumor and germline DNA from patient 984 with masked hypodiploidy is represented. The imbalanced peaks detected in leukemia cells are indicated by red arrows.

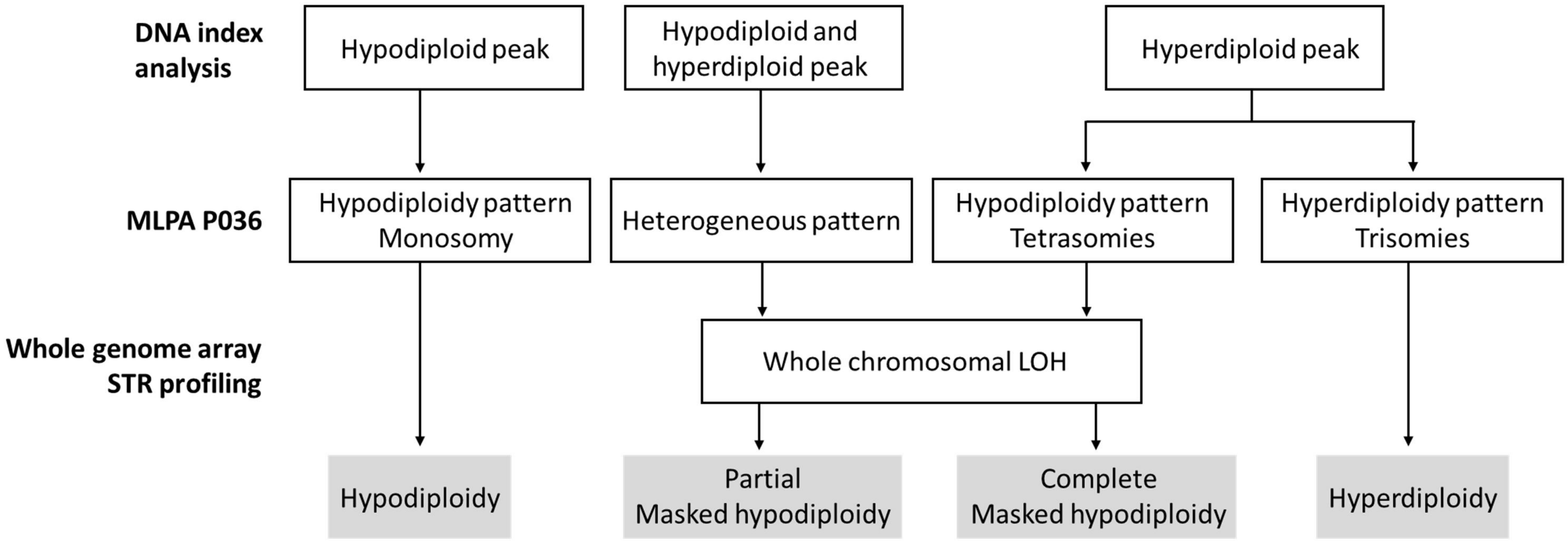

Supplementary Figure S6. Flowchart for distinguishing masked hypodiploidy from high hyperdiploidy.

(a)

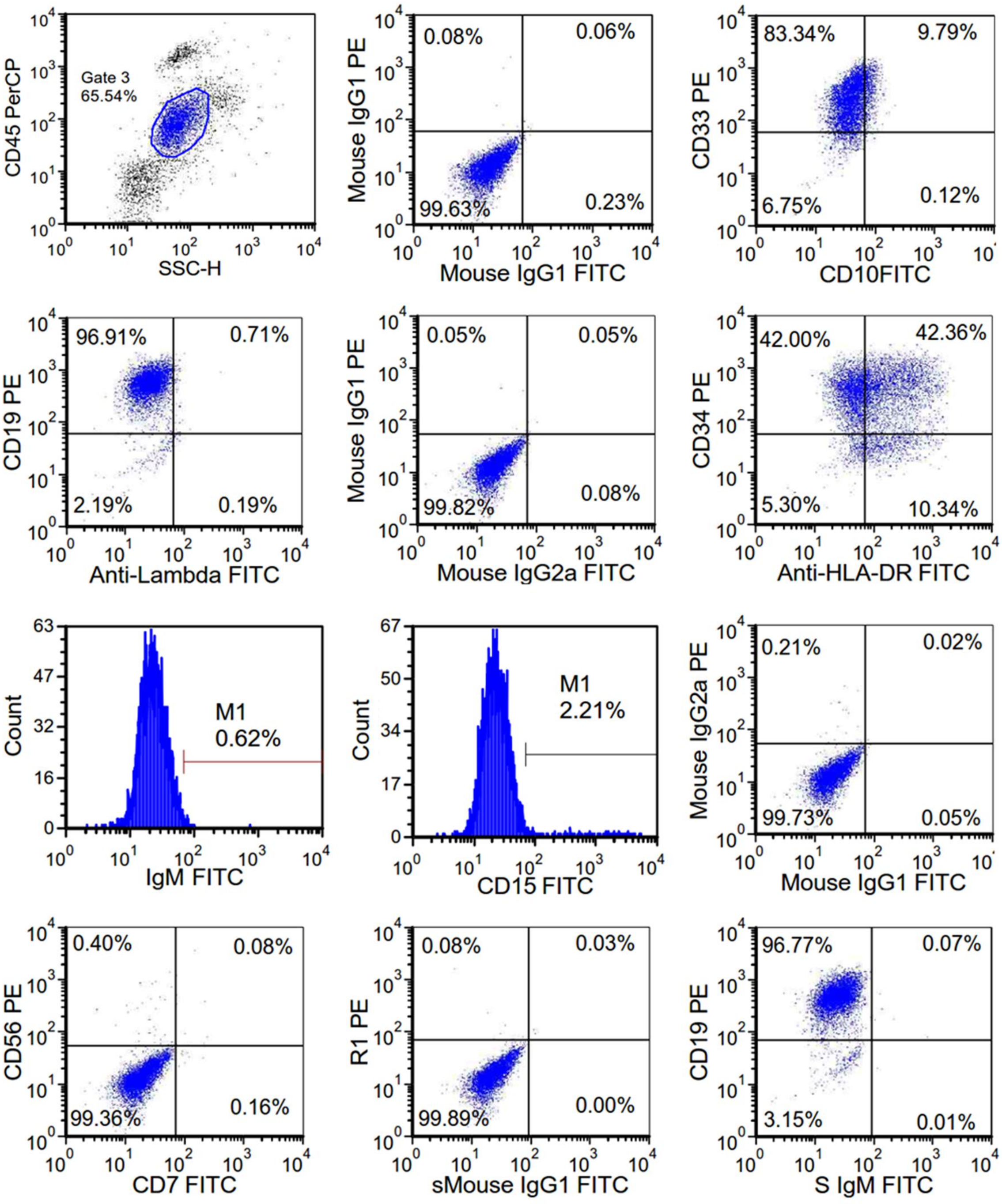

(b)

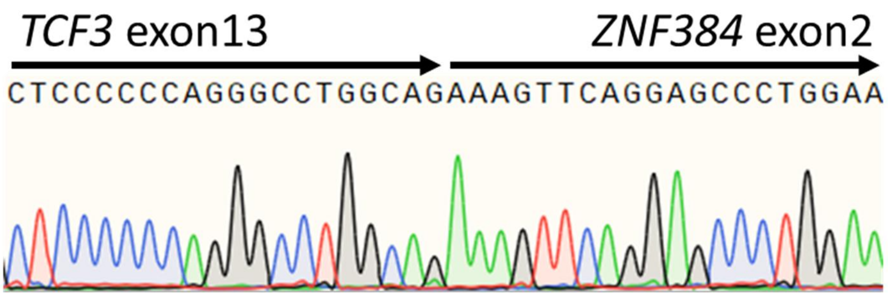

Supplementary Figure S7. Analysis of a case with *TCF3-ZNF384* case.

(a) The immunophenotype shows CD19+, CD10-, CD13+ and CD33+ B-cell ALL. (b) The *TCF3-ZNF384* fusion gene is confirmed by RT-PCR and Sanger sequencing.
